# Grafting Cell‐Penetrating Poly(disulfide)s to Substrates of Interest: Dynamic Covalent Bioconjugation for Traceless Delivery

**DOI:** 10.1002/anie.202517229

**Published:** 2025-11-06

**Authors:** Michael Cognet, Giacomo Renno, Filipe Coelho, Naomi Sakai, Stefan Matile

**Affiliations:** ^1^ Department of Organic Chemistry University of Geneva Geneva Switzerland; ^2^ National Centre of Competence in Research (NCCR) Molecular Systems Engineering Basel Switzerland

**Keywords:** Bioconjugation, Cellular uptake, Dynamic‐covalent chemistry, Polymer chemistry, Protein delivery

## Abstract

Although increasingly understood and appreciated, thiol‐mediated uptake (TMU) remains underused because practical traceless tags that solve daily delivery problems are not yet available. The most popular cell‐penetrating poly(disulfide)s (CPDs) were initially introduced as traceless tags that could be grafted from any thiol‐containing substrate of interest (SOI) in situ and would depolymerize in the cytosol right after uptake. This approach was operational but not ideal for solving practical problems because the concentrations of SOIs >30 µM needed in neutral water are above those acceptable in most biological studies. Here, we report that CPD grafting‐to SOIs, rather than grafting‐from, provides access to dynamic covalent cysteine bioconjugation with protein concentrations down to 50 nM, which is more than 600 times below standard grafting‐from CPD chemistry. With rate constants up to 1500 M^−1^s^−1^, CPD grafting‐to is as fast as the record covalent cysteine bioconjugation (heteroaromatic sulfones), in the range of the best bioorthogonal reaction (IEDDA), and 3000 times faster than cystine grafting‐to. Experimental evidence for CPD grafting to probes, peptides and proteins with one, two, or several proximal thiols, efficient TMU of their conjugates, and the cytosolic release of functional SOIs, such as fluorescent antibodies against the nuclear pore complex, supports the discovery of operational traceless TMU tags, at last.

## Graphical abstract

Grafting‐to rather than the grafting‐from strategy envisioned 10 years ago is shown to make cell‐penetrating poly(disulfide)s compatible with dynamic covalent bioconjugation at high dilution (50 nM) and top rates (1500 M^−1^s^−1^), genetic engineering and transduction domains, that is traceless tags for protein delivery, including intact functional anti‐NPC antibodies.

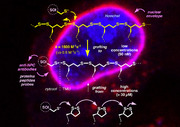

Chemical control over the entry into cells is of utmost importance to science and society for both activation (drug delivery) and inhibition (drug discovery), but also to enable basic science and elucidate cellular processes, for example, with synthetic proteins.^[^ ^1^, ^2^, ^3^, ^4^, ^5^, ^6^, ^7^, ^8^, ^9^, ^10^, ^11^, ^12^, ^13^, ^14^, ^15^, ^16^ ^]^ To tackle this challenge, we have introduced cell‐penetrating poly(disulfide)s (CPDs) as conceptually new way to penetrate cells (Figure 1).^[^ ^17^, ^18^ ^]^ CPDs^[^ ^1^, ^2^, ^3^, ^4^, ^17^, ^18^, ^19^, ^20^, ^21^, ^22^ ^]^ and related systems^[^ ^6^, ^13^, ^14^, ^15^, ^23^ ^]^ take advantage of two mechanistically distinct delivery strategies, i.e., thiol‐mediated uptake (TMU) centered around dynamic‐covalent cascade exchange with cellular proteins^[^ ^16^, ^24^, ^25^ ^]^ and arginine‐rich cell‐penetrating peptides (CPPs) based on noncovalent binding to anionic lipids.^[^ ^6^, ^7^, ^8^, ^9^, ^26^, ^27^ ^]^ Since their introduction, CPDs have been optimized and applied broadly, also in vivo.^[^ ^1^, ^2^, ^3^, ^4^, ^18^, ^19^, ^20^, ^21^, ^22^, ^28^, ^29^, ^30^, ^31^, ^32^, ^33^, ^34^ ^]^ They attract such interest because they are more active than mono‐ and oligomeric TMU systems,^[^ ^5^, ^25^, ^35^, ^36^, ^37^, ^38^ ^]^ and their intracellular depolymerization releases unmodified substrates of interest (SOIs) in the cytosol (Figure 1c).

**Figure 1:**
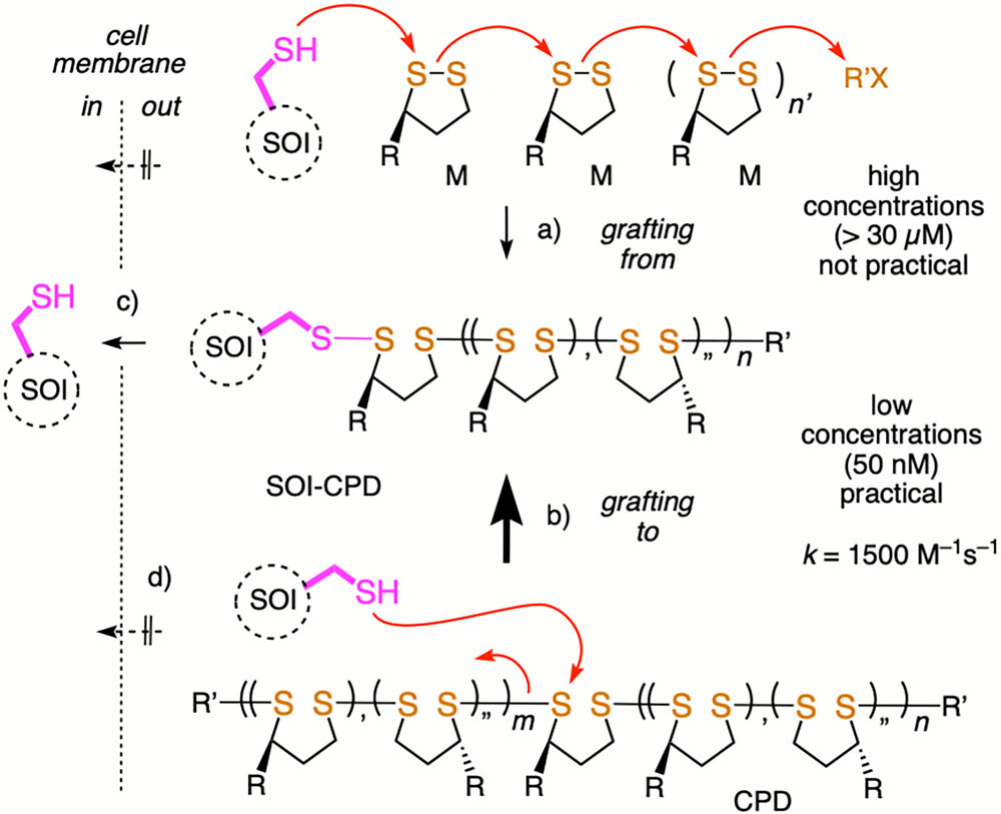
Grafting‐to b) rather than grafting‐from a) dynamic covalent CPD bioconjugation for the c) delivery of unmodified substrates of interest (SOIs) into the cytosol is promising in practice because high rates enable dynamic covalent traceless tagging in situ at biorelevant low concentrations, while d) noncovalent formal polyplexes are inactive (M, monomer; *n’* + *m’*/*n”* + *m”* = 3:2;^[^ ^22^ ^]^ R, R’: see Figure 2).

The critical weakness that prevents the development of CPDs as generally applicable tools for solving practical delivery problems is the substrate conjugation step. The ideal delivery tool is a traceless tag that can be attached to the SOI at the low concentrations usually required for biological studies. Initially inspired by surface polymerization chemistry, CPDs were prepared by ring‐opening polymerization of monomer M, which consists of lipoic acid and arginine, grafted from the SOI with a thiol as an initiator (Figure 1a).^[^ ^18^, ^40^ ^]^ Despite much effort to improve,^[^ ^3^, ^32^ ^]^ practical versions of this grafting‐from approach still require SOI concentrations from 30 µM to 4 mM in neutral buffer.^[^ ^2^, ^41^, ^42^, ^43^ ^]^ For various reasons, classical bioconjugation approaches have not yet led to popular use of CPDs by nonexperts in the community either.^[^ ^1^, ^44^, ^45^ ^]^

For bioconjugation at high dilution, the rate constant of the reaction is decisive. The onset of fast bioconjugation has been placed at *k* = 10 M^−1^s^−1^ in neutral water at room temperature, which translates to 97% conversion of 10 µM SOI in 1 h with 10 equiv of reagent.^[^ ^46^ ^]^ Among bioorthogonal classics, Staudinger ligations (*k* ∼ 10^−3^ M^−1^s^−1^) and SPAAC (*k* ∼ 10^−3^ – 1 M^−1^s^−1^) are below, while IEDDA (*k* ∼ 1 – 10^6^ M^−1^s^−1^) is mostly above this threshold and approaches enzymatic bioconjugation kinetics.^[^ ^47^ ^]^

For cysteine (Cys) bioconjugation, rates depend strongly on conditions and the nature of the Cys involved, particularly N‐terminal ones operate with different chemistry.^[^ ^48^, ^49^ ^]^ For average Cys, iodoacetamides have been reported to react at 0.6 M^−1^s^−1^, maleimides at 100 M^−1^s^−1^,^[^ ^50^ ^]^ and the best covalent reagents, heteroaromatic sulfones, at 1650 M^−1^s^−1^,^[^ ^46^, ^51^ ^]^ while vinyl thianthrenium salts did not exceed 310 M^−1^s^−1^, which in practice resulted in average 10–15 µM protein concentrations.^[^ ^52^ ^]^ Here, we demonstrate that grafting CPDs to SOIs, rather than grafting‐from, provides access to SOI‐CPD bioconjugates with *k * ∼ 1500 M^−1^s^−1^ and SOI concentrations as low as 50 nM (Figure 1b), which is more than 600 times below the concentrations reported^[^ ^2^, ^41^, ^42^, ^43^ ^]^ for grafting‐from CPD chemistry (Figure 1a).

During an inhibitor screening to identify TMU exchange networks,^[^ ^25^, ^39^, ^53^ ^]^ we noticed that CPDs **1** activate, rather than inhibit, TMU of certain cyclic disulfides. This surprise finding suggested that CPDs exchange with these SOIs under biological conditions. This hypothesis was verified first using simple fluorescent Cys **2** as a model SOI (Figure 2a and Scheme S2). Fluorophore‐free CPDs **1** were prepared with an average molecular weight of *M*_n_ = 35 ± 5 kDa, corresponding to *n* = 80 ± 10 monomers, and a dispersity of *Ð* = 1.3 ± 0.1 (Figure S3). Grafting of CPD **1** to Fl‐Cys **2** in neutral buffer was confirmed by the appearance of a fluorescent polymer band in the size‐exclusion chromatogram (SEC, Figure 2c (A)). Unchanged SECs of the thiol‐free control **3** treated with **1** (Figure 2c (B)) confirmed the formation of conjugate **2–1** through disulfide exchange between **2** and **1**. The *M*_n_ = 20 ± 1 kDa of **2–1** with unchanged *Ð* = 1.2 ± 0.1 suggested that grafting CPDs to SOIs with single thiols occurs in the middle of the polymer (Figure 2c (A)). Central rather than terminal exchange was preferred to benefit from maximal SOI–polymer interactions and possible disulfide activation^[^ ^54^ ^]^ from CPD folding. The slightly higher acidity of benzyl thiol was thus unable to direct the exchange to the termini, and more acidic thiols like thiophenols would produce macrocyclic CPDs.^[^ ^55^ ^]^

**Figure 2:**
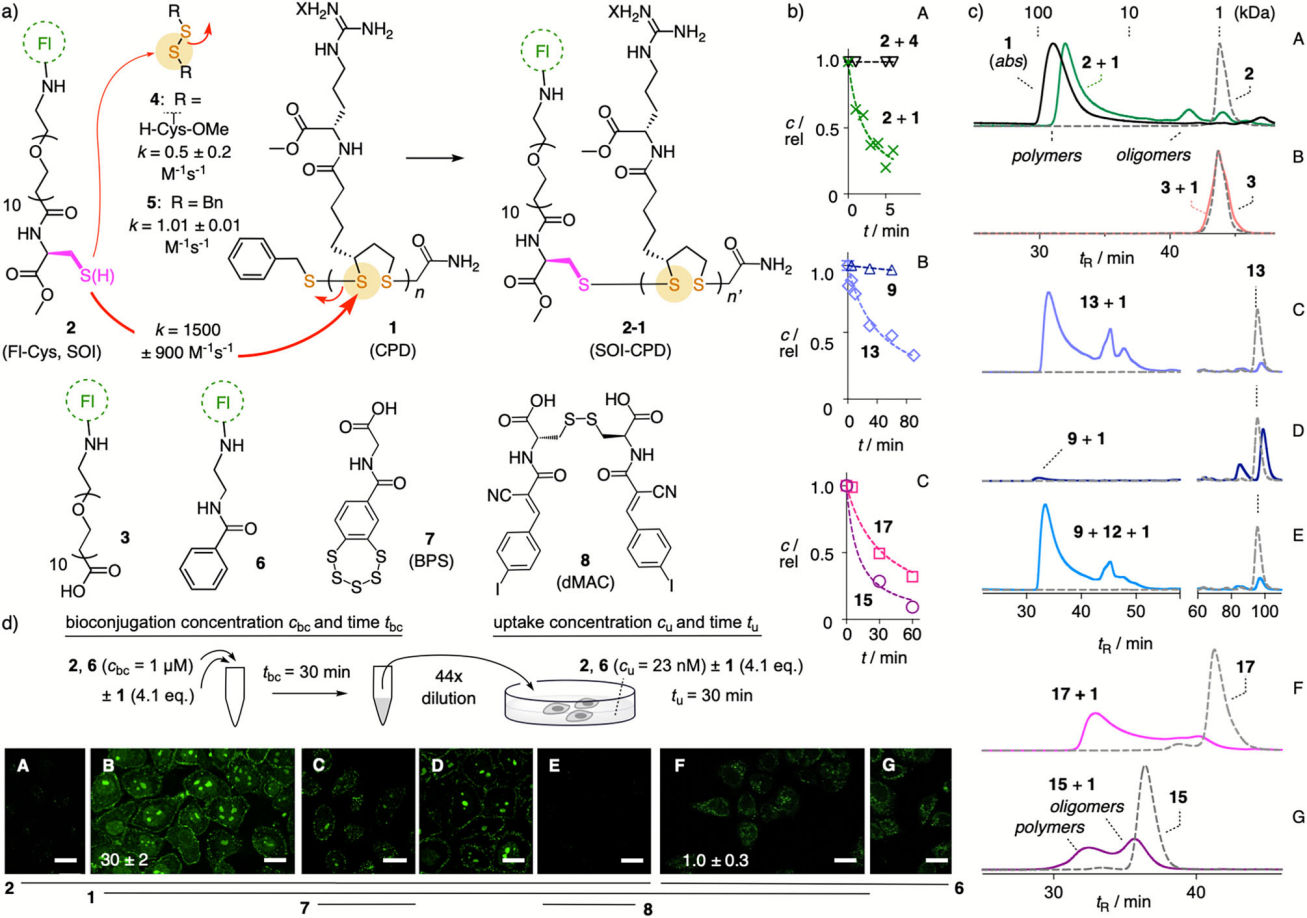
a)–c) Grafting‐to CPD bioconjugation and d) uptake of SOI **2** with a single thiol, with controls **3**–**6** and inhibitors **7** and **8** (Fl = FITC, *n* = 80). b) SOI concentration as a function of time during exchange with **1** (∼1 equiv, all except ▽) and **4** (▽, 1 equiv) in DPBS:CH_3_CN 7:3, pH 7.4, rt, for A) **2** (X, ▽, 4 µM), B) **9** (□), **13** (◯, both 1.5 mM), C) **15** (□) and **17** (◯, both 750 µM), with fit to second‐order kinetics (RP‐HPLC kinetics; for **9**, **13**, **15**, and **17**, see Figure 3). c) Size‐exclusion chromatograms (SECs) of CPD **1** (A, black solid, absorption) and SOIs before (dashed) and after (solid) CPD bioconjugation for A) **2** versus B) **3** (bioconjugation concentration *c*_bc_ = 1 µM, 4.1 equiv **1**, bioconjugation time *t*_bc_ = 30 min), and for C) **13**, D) **9**, E) **9** plus **12**, F) **17**, and G) **15** (*c*_bc_ = 750 µM, 1.2 equiv **1**, *t*_bc_ = 60 min). d) Confocal laser scanning microscopy (CLSM) images of HK cells incubated with **2** (A–E) and **6** (F and G; uptake concentration *c*_u_ = 23 nM, uptake time *t*_u_ = 30 min (C–E: *t*_u_ = 20 min), all in L‐15), without (A and G) and after bioconjugation (B–F, *c*_bc_ = 1 µM, 4.1 equiv **1**, *t*_bc_ = 30 min), without or with uptake inhibition by preincubation with **7** (C, inhibitor concentration *c*_i_ = 5 µM) or **8** (E, *c*_i_ = 50 µM, inhibitor treatment time *t*_i_ = 60 min). Comparable intensities: A versus B, C–E, F versus G; intensity changes ± **1**: *I*/*I*_0_ ± SD (B, F; *I*_0_ from A, G; SD from technical triplicates; statistical analysis in Figure S13); scale bars: 20 µm; concentrations of **1** represent polymer concentrations.

Grafting of **1** to **2** occurred with a rate constant of *k* = 1500 ± 900 M^−1^s^−1^ per polymer (mean ± SD of experimental replicates, or 9 ± 6 M^−1^s^−1^ per monomer, which is an irrelevant value for the assessment of the bioconjugation reaction,^[^ ^54^ ^]^ Figure 2b (A)). This rate was in the range of the best bioorthogonal (IEDDA)^[^ ^47^ ^]^ and covalent Cys bioconjugation (*k *= 1650 M^−1^s^−1^).^[^ ^46^, ^51^ ^]^ Compared to the 1500 M^−1^s^−1^ for CPD **1**, grafting of cystine dimethyl esters **4** with mildly activated disulfides was with *k *= 0.5 ± 0.2 M^−1^s^−1^ 3000 times slower (Figures 2b (A) and S9). Dibenzyldisulfides **5** were with *k *= 1.01 ± 0.01 M^−1^s^−1^ only twice as fast and still 1500 times slower than CPDs, which was consistent with the absence of selectivity for bioconjugation at the CPD termini (Figures 2c (A) and S9).

The high CPD bioconjugation rates were likely to originate from unspecific noncovalent SOI–polymer interactions, particularly repulsion‐driven ion pairing^[^ ^9^, ^27^ ^]^ but also hydrogen bonding and, perhaps, CPD folding. Such weak but additive protein–polymer interactions have been used for noncovalent uptake of formal polyplexes,^[^ ^2^, ^26^ ^]^ an approach that was not validated by the systems used in this study (see below).

CPD‐conjugate **2–1** was prepared by reacting SOI **1** at a bioconjugation concentration *c*_bc_ = 1 µM with 4.1 equiv CPD **1** for *t*_bc_ = 30 min and then diluted to an uptake concentration *c*_u_ = 23 nM (Figure 2d). Conjugate **2–1** was not toxic at this concentration (Figure S14) and entered HeLa Kyoto (HK) cells efficiently (Figure 2d (B)). Compared to **2** without **1** (Figure 2d (A)), fluorescence intensity increased 30‐times. CPD **1** did not increase TMU of control **6**, demonstrating that dynamic covalent bonds between CPD and SOI are needed (Figure 2d (F and G)). Selective inhibition by TMU probes **7** ^[^ ^56^ ^]^ and **8**,^[^ ^57^ ^]^ supported that **2–1** enters cells along distinct TMU exchange networks (Figures 2d (C–E)) and S17).

To elaborate on SOIs with two proximal thiols, the fluorescent asparagusic acid derivative Fl‐AspA **9** ^[^ ^58^ ^]^ with a strained disulfide was considered first. Addition of CPD **1** enabled TMU (Figure 3a (A–C)) but did not produce the SEC peak of CPD conjugate **9–1** (Figure 2c (D)). This discrepancy implied that **9** exchanged first with extracellular thiols **10** to produce the thiol in **11** that is needed to proceed to CPD‐conjugates **9–1**. Indeed, in the presence of the biomimetic Cys **12**, the **9–1** peak appeared in the SEC (Figure 2c (E)).

**Figure 3:**
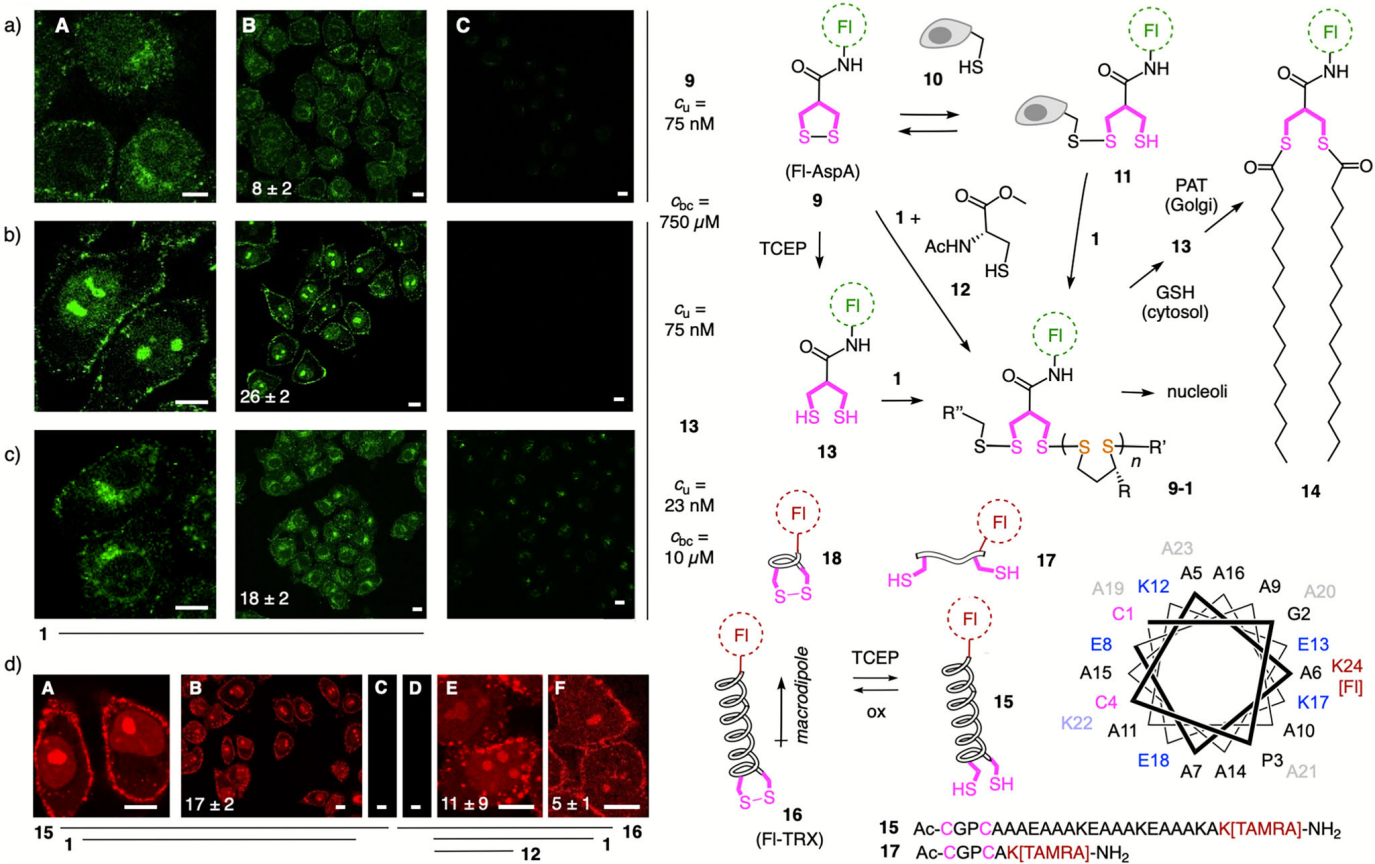
Grafting‐to CPD bioconjugation and uptake of SOIs with two proximal thiols from a)–c) strained cyclic disulfides and d) α‐helical TRX mimics. a)–c) CLSM images of HK cells incubated with **9** (a, *c*_u_ = 75 nM) and **13** (b, *c*_u_ = 75 nM; c, *c*_u_ = 23 nM; *t*_u_ = 30 min), without (C) and after bioconjugation (A, B; a, b, *c*_bc_ = 750 µM, 1.3 equiv **1**; c, *c*_bc_ = 10 µM, 4.1 equiv **1**; *t*_bc_ = 1 h). d) Same for **15** (A–C) and **16** (D–F, *c*_u_ = 75 nM, *t*_bc_ = 30 min), without (C and D) and after bioconjugation with **1** (A and B, E and F; *c*_bc_ = 750 µM, 1.25 equiv **1**, *t*_bc_ = 1 h), without (A–D, F) or with **12** (1 equiv, E), comparable intensities: B and C, D and E; intensity changes ± **1**: *I*/*I*_0_ ± SD (B, E, F, *I*_0_: C, D; statistical analysis in Figure S13); scale bars: 10 µm.

Addition of CPD **1** to reduced Fl‐AspA **13** gave the same SEC **9–1** peak (Figure 2c (C)), TMU increased 26‐times and intracellular localization shifted from the Golgi to the nucleoli (Figure 3a versus Figure 3b; like **2–1**, Figure 2d (B and D)). A 75‐fold dilution of the bioconjugation product mixture caused a clean relocalization from nucleoli to the Golgi (Figure 3b versus Figure 3c; Figures S15 and S16). CPDs like **1** and CPPs track nucleoli^[^ ^18^, ^59^, ^60^ ^]^ and Fl‐AspA **9** is a Golgi tracker, where it is palmitoylated and immobilized as amphiphile **14** (Figure 3).^[^ ^58^ ^]^ Relocalization with changing concentrations thus implied that cytosolic CPD depolymerization liberating Golgi‐tracking **9** is in kinetic competition with the escape of intact CPD‐conjugates **9–1** into the nucleus.

Additional oligomer peaks in the SEC besides the main **9–1** polymer peak suggested that the second thiol in AspA **13** shortens CPDs that are attached to the first one, including macrocyclization (Figure 2c (C)).^[^ ^31^, ^55^, ^61^, ^62^ ^]^ The *k* = 0.26 ± 0.03 M^−1^s^−1^ for dithiol **13** (Figure 2b (B)), much slower than monothiol **2** but still better than Staudinger ligations and as good as SPAAC,^[^ ^47^ ^]^ was consistent with decelerating competition from the second thiol.

To elaborate on peptidic dithiols as potential transduction domains, a fluorescent thioredoxin (TRX)^[^ ^63^, ^64^, ^65^, ^66^ ^]^ mimic was designed (Figure 3d).^[^ ^67^ ^]^ In α helix^[^ ^68^ ^]^ **15**, the CGPC thiols should be acidified by the helical macrodipole and N‐terminal hydrogen bonds,^[^ ^63^, ^64^, ^65^, ^66^ ^]^ while the macrocyclic disulfide in oxidized **16** should be strained by topological mismatch with helix stapling.^[^ ^69^ ^]^ A formal redox potential of −320 mV of disulfide exchange equilibrium^[^ ^70^ ^]^ suggested that ring tension in **16** is weaker than in lipoic acid monomers (−290 mV) and AspA **9** (−270 mV), while a drop to −340 mV upon thermal denaturation confirmed its existence (Figures S21, S23, and S25). The *k* = 1.0 ± 0.4 M^−1^s^−1^ for grafting CPD **1** to TRX mimic **15** was faster than *k* = 0.26 ± 0.03 M^−1^s^−1^ for AspA **13** and also *k* = 0.5 ± 0.2 M^−1^s^−1^ for the helix‐free CXXC control **17** (Figure 2b (C and B)). Compared to AspA **13**, N‐terminal CXXC **15** thus exchanged faster with disulfides despite a higher propensity for ring closure. Nearly full consumption of **15** (Figure 2b (C)) implied that the main peak just before the high molecular weight substrate peak in the SEC originates from bioconjugated short CPD oligomers, which implied substantial macrocyclization with the second reactive thiolate firmly positioned and activated at the N terminus of the α helix (Figure 2c (G)). Negligible oligomer peaks with the disordered control **17** confirmed the importance of preorganization for CPD macrocyclization on dithiol **15** (Figure 2c (F)).

Like SEC, uptake results for TRX **15** were consistent with those of Fl‐AspA **9**. CPD bioconjugation increased the inhibitable (Figure S20) TMU of dithiol **15** 17‐times and that of disulfide **16** 5‐times without and 11‐times in the presence of Cys **12** (Figure 3d). Less efficient CPD bioconjugation shifted localization of **15** from the nucleus, particularly nucleoli, toward diffuse labeling of the cytosol due to the absence of a specific intracellular target (like the Golgi for AspA **9**; Figure 3d (F versus A)). Similar uptake found for the disordered short controls **17** and **18** implied that the special characteristics of the N‐terminal α‐helical CXXC compensated for the more demanding TMU of larger substrates like **15** and **16** (Figure S10).

With two proximal thiols being overall less effective, the simplest TMU transduction domain for genetic engineering remained a single Cys. The S147C GFP mutant **19** was chosen to ensure comparability with the recently reported covalent vinyl thianthrenium bioconjugation with maximal 310 M^−1^s^−1^, which is slower than CPD bioconjugation (Figure 4).^[^ ^52^ ^]^ Isosteric S147C mutation installs one surface‐exposed Cys at the edge of the β barrel with one R168 as only nearby charge. HK cells were incubated with *c*_u_ = 23 nM of **19** after conjugation at *c*_bc_ = 1 µM with 4.1 equiv CPD **1** for *t*_bc_ = 1 h in DPBS. The CPD tag **1** caused a 25‐fold increase of the inhibitable (Figure 4a (E–G)) TMU of the protein (Figure 4a (A and B)). In contrast, CPD **1** did not increase the uptake of nonmutated GFP **20**, confirming that noncovalent interactions between protein and CPD were insufficient to enable TMU (Figure 4a (C and D)).

**Figure 4:**
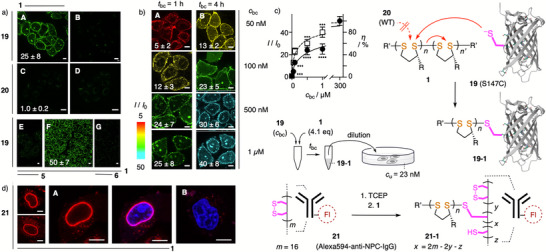
Grafting‐to CPD bioconjugation and uptake of proteins with a)–c) genetically engineered single‐thiol transduction domains (GFP) and d) multiple native vicinal thiols from disulfide reduction (antibodies). a) CLSM images of HK cells incubated with S147C GFP mutant **19** (A, B, *c*_u_ = 23 nM; E–G, *c*_u_ = 34 nM) or WT GFP **20** (C, D, *c*_u_ = 23 nM, *t*_u_ = 30 min), without (B and D) and after bioconjugation (A, C; *c*_bc_ **=** 1 µM, 4.1 equiv **1**; E–G; *c*_bc_ **=** 340 µM, 1.2 equiv **1**; *t*_bc_ **=** 1 h), without (A–D, F) and with uptake inhibition by preincubation with **7** (E, *c*_i_ = 5 µM) or **8** (G, *c*_i_ = 50 µM, *t*_i_ = 60 min). Image intensities are comparable within the series for A and B, C and D, E and G; intensity changes ± **1**: *I*/*I*_0_ ± SD (technical triplicates). b) Same for **19** (*c*_u_ = 23 nM) after bioconjugation with *c*_bc_ = 50, 100, 500, and 1000 nM (4.1 equiv **1**, top down) for (A) *t*_bc_ = 1 h and (B) *t*_bc_ = 4 h (scale bar = 10 µm). Intensities are color‐coded based on changes ± **1**: *I*/*I*_0_ ± SD (technical triplicates). S147C mutant **19** solution contained DTT (2 equiv) and was used without purification. c) Dependence of uptake increase *I*/*I*_0_ of GFP **19** on *c*_bc_ and *t*_bc_ with CPD **1** (∼1.4 equiv), with the results of nonparametric two‐tailed *t*‐tests (*p >*0.1234: ns, <0.0002: ***, <0.0001: ****; complete *t*‐tests, Figure S13); error bars represent SEM. d) CLSM images of HK cells incubated with Alexa594‐anti‐NPC‐IgG **21** (*c*_u_ = 23 nM, 4.1 equiv **1**) without (B) and after CPD bioconjugation (A, *c*_bc_ = 4 µM, 1. 4 µM TCEP, 5 min, 2. 4.1 equiv **1**, *t*_bc_ = 4 h). A: Three examples (left) and co‐labeling with Hoechst 33342 (blue, nuclei, right), scale bars = 10 µm.

Using TMU as a readout, the bioconjugation reaction was evaluated at a lower *c*_bc_ of **19** while keeping the CPD equivalent and *c*_u_ constant (Figure 4b). A 13‐fold increased TMU remained well detectable for protein **19** tagged with CPD **1** at concentrations as low as *c*_bc_ = 50 nM after a conjugation time of *t*_bc_ = 4 h. Coinciding with cytosol‐to‐nucleus relocalization as for other SOIs (Figure 4a,b), increasing TMU with increasing *c*_bc_ of GFP **19** saturated at a factor of 50 (Figure 4c). Independence of maximal TMU on *t*_bc_ confirmed that this saturation behavior reveals conjugation efficiency quantitatively. For GFP **19** with 4.1 equiv CPD **1**, genetically engineered surface‐accessible single Cys transduction domains were characterized by *c*_bc_^50^ = 0.2 ± 0.04 µM for 50% and *c*_bc_^95^ ∼ 5 µM for full bioconjugation within *t*_bc_ = 4 h.

Despite lower bioconjugation efficiency, native vicinal thiols from reduced disulfides could be used for traceless tagging of the monoclonal antinuclear pore complex (NPC) IgG modified with Alexa Fluor 594 (Alexa594‐anti‐NPC‐IgG)^[^ ^7^ ^]^ **21**. After reduction with TCEP and bioconjugation with CPD **1**, the conjugate **21–1** labeled the nuclear envelope, demonstrating cytosolic delivery of functional antibodies (Figure 4d (A)). The inability of CPD‐free antibodies **21** to penetrate cells confirmed that traceless tagging by grafting‐to of CPDs is compatible with the cytosolic delivery of large functional SOIs under biologically practical conditions (Figure 4d (B)).

The discovery of dynamic covalent grafting‐to bioconjugation of cell‐penetrating poly(disulfide)s as traceless tags is important because it can make TMU useful to solve daily delivery problems in the community. The likely origin of high bioconjugation rates from noncovalent protein–polymer interactions and, perhaps, polymer folding suggests that they can be further increased by the rational design of advanced transduction domains on the protein side and modern multicomponent CPDs^[^ ^1^, ^2^, ^3^, ^4^, ^18^, ^19^, ^20^, ^21^, ^22^, ^28^, ^29^, ^30^, ^31^, ^32^, ^33^, ^34^ ^]^ on the polymer side.

## Supporting Information

Experimental details.

## Conflict of Interests

AspA Golgi trackers have been commercialized by Spirochrome.

## Supporting information



Supporting Information

## Data Availability

The data that support the findings of this study are openly available in zenodo at https://doi.org/10.5281/zenodo.17189596.
