# Supplementary material for: Grafting Cell‐Penetrating Poly(disulfide)s to Substrates of Interest: Dynamic Covalent Bioconjugation for Traceless Delivery
Source: Angew Chem Int Ed Engl. 2025 Nov 6;65(1):e17229. doi: 10.1002/anie.202517229 (PMC12759256; doi:10.1002/anie.202517229)
Supplement: Supplementary file 1 — Supporting Information [file ANIE-65-e17229-s001.pdf]

Supporting Information

Grafting Cell-Penetrating Poly(disulfide)s to Substrates of Interest: Dynamic Covalent Bioconjugation for Traceless Delivery

Michael Cognet,[a-c] Giacomo Renno,[a-c] Filipe Coelho,[a,b] Naomi Sakai[a,b]
and Stefan Matile*[a,b]

[a]Department of Organic Chemistry, University of Geneva, Geneva, Switzerland

[b]National Centre of Competence in Research (NCCR) Molecular Systems Engineering, Basel, Switzerland

[c]These two authors contributed equally to this study.

*E-mail: stefan.matile@unige.ch

Table of Contents

1. Materials and Methods S4
2. Synthesis S7
    2.1. Synthesis of CPDs S7
    2.2. Synthesis of SOIs S9
    2.3. Synthesis of TMU Inhibitors S13
3. General Procedures for Grafting-to S13
    3.1. Stock Solutions and Buffers Used Throughout the Study S13
    3.2. General Procedure for Grafting to Small Molecules and IgG S14
    3.3. General Procedure for Grafting to sfGFP Mutant **19** S14
4. Polymer Characterization by SEC S15
    4.1. Characterization of CPDs S15
    4.2. Characterization of Grafting-to Reaction Mixtures S16
        4.2.1. Grafting CPDs to Fl-Cysteine **2** and **3** S16
        4.2.2. Grafting CPDs to Cyclic Disulfides **9**, **16**, **18** and Analogues S16
5. Bioconjugation Kinetics S19
    5.1. Preparation of Reaction Mixtures S19
    5.2. Data Analysis S21
    5.3. Exchange of **2** with Disulfides **4** and **5** S22
6. Cell Culture S23
7 Cellular Uptake S24
    7.1. General Procedure for CLSM Experiments S24
    7.2. Data Analysis S24
    7.3. Cellular Uptake of Grafted-to Bioconjugates S24
    7.4 Cell Viability Assay S30

7.5 Colocalization Experiments S31

7.5.1. Colocalization of **9-1** with Golgi Tracker **32** S31

7.5.2. Nucleoli Staining S33

8 Inhibition Assay S33

8.1. General Procedure S33

9. Measurement of Reduction Potentials S36

9.1. General Procedure S36

9.2. Effect of Helix Secondary Structure on the Redox Potential S39

10. Supporting References S41

11. NMR Spectra S43

1. Materials and Methods

As in reference [S1], reagents for synthesis were purchased from Sigma-Aldrich, FluoroChem, and TCI. Peptide **33** (DP-CGPCAAAEAAAKEAAAKEAAAKAK[N_3]-NH_2) was custom synthesized by GenScript Biotech (purity > 95%). Peptides **18** (Ac-CGPCAK[TAMRA]-NH_2) and **16** (Ac-CGPCAAAEAAAKEAAAKEAAAKAK[TAMRA]-NH_2) were custom synthesized by CASLO ApS (96% of purity). sfGFP S147C **19** and mNeonGreen **20** were produced at Protein Biochemistry Platform, Faculty of Medicine, University of Geneva, Switzerland. Alexa594-anti-NPC-IgG (mAb414) **21** was purchased by BioLegend. Dulbecco's PBS (calcium/magnesium-free, DPBS, 14190094), DMEM (11965092), FluoroBrite DMEM (GlutaMAX, 4.5 g/L D-glucose, pyruvate, without phenol red, A1896701), and Leibovitz's L15 medium (without phenol red, 21083027) were obtained from Thermo Fisher Scientific, as well as fetal calf serum, TrypLE Express enzyme, V96-MicroWell plates, Nunclon™ Delta Surface 96-well sterile plates, and Hoechst 33342 (HOE, H3570). Cell-Titer Blue G8080 kit for cell viability determination was purchased from Promega. 35 mm glass-bottom culture dishes were purchased from MatTek Dishes. Column chromatography was carried out on silica gel (SilicaFlash® P60, SILICYCLE, 230–400 mesh). Flash column chromatography was performed on a Biotage Selekt system. Analytical thin-layer chromatography (TLC) was performed on silica gel 60 F254 (Merck, 0.2 mm). Fluorescence and UV-vis absorption measurements were performed on a FluoroMax-4 spectrofluorometer and JASCO V-650 spectrometer, respectively, equipped with a stirrer and temperature controller. Fluorescence spectra were corrected for lamp intensity fluctuations, background and the wavelength-dependent response function of the detector. Fluorescence data for viability measurements were measured using a MicroWell plate reader MicroMax 384 attached to FluoroMax-4. Circular dichroism (CD) spectra were obtained using JASCO J-815 spectropolarimeter and were reported as extremum wavelength λ in nm ($\Delta\varepsilon$ in $M^{-1}cm^{-1}$). IR

spectra were recorded on a Perkin Elmer Spectrum Two FT-IR spectrometer (ATR, Golden Gate) and were reported as wavenumbers v in cm^{-1} with band intensities indicated as s (strong), m (medium), w (weak), br (broad). ^{1}H, ^{13}C NMR spectra were recorded (as indicated) on a Bruker 300 MHz, 400 MHz, or 500 MHz spectrometer, at 25 °C, and were reported as chemical shifts (δ) in ppm relative to TMS (δ = 0). Spin multiplicities were reported as a singlet (s), doublet (d), triplet (t) quartet (q), quintet (quint), doublet of doublets (dd), triplet of doublets (td) with coupling constants (J) given in Hz, broad singlet (br s) or multiplet (m). ^{1}H and ^{13}C resonances were assigned with the aid of additional information from 1D and 2D NMR spectra (H,H-COSY, DEPT 135, HSQC and HMBC). LC-MS analyses were performed on Advion Avant® UHPLC system equipped with a Thermo C18 Hypersil GOLD column (50 × 2.1 mm, 1.9 µm particles size) with Advion Expression® CMS in ESI mode. ESI-HRMS was measured on Xevo G2-S Tof (Waters). All mass data were reported as mass-per-charge ratio m/z ([assignment]). Size-exclusion chromatography (SEC) purification and analyses were performed using a JASCO LC-2000 Plus system equipped with a quaternary pump (JASCO PU2089), an absorbance (JASCO UV-2077 Plus) and a fluorescence (JASCO FP-2020 Plus) detectors. The chromatographic column used was a Superdex 75 10/300 GL. Samples were run with a 0.4 mL/min flowrate from 0 to 40 min, then 0.6 mL/min from 41 to 120 min in isocratic conditions, with a mixture of 30% CH_3CN in 0.1 M acetate buffer pH = 6.5 as an eluent. Absorbance was measured at 220, 255, and 330 nm, while fluorescence parameters were tuned depending on the fluorophore (FITC: λ_{ex} = 490 nm, λ_{em} = 520 nm; TAMRA: λ_{ex} = 552 nm, λ_{em} = 578 nm). High pressure liquid chromatography (HPLC) analyses were performed using JASCO LC-4000 Plus system equipped with a quaternary pump (PU-4580 with LG-4580), and a PDA detector (MD-4010). The chromatographic column used was a Phenomenex Gemini 5 µm C18 110 Å, 150×3.00 mm column. Confocal laser scanning microscopy (CLSM) was performed on Leica SP8, equipped with 63x oil immersion objective.

Abbreviations. CLSM: Confocal laser scanning microscopy; COI: Compound of interest; CPD: Cell-penetrating poly(disulfide); DIPEA: *N*,*N*-Diisopropylethylamine; DMF: *N*,*N*-Dimethylformamide; DMSO: Dimethylsulfoxide; DPBS: Dulbecco's phosphate buffered saline; DTT: Dithiothreitol; EDC: 1-ethyl-3-(3-dimethylaminopropyl)carbodiimide; EtOAc: Ethyl acetate; FA: Formic acid; FDMEM: FluoroBrite™ Dulbecco's modified eagle medium; Fl: Fluorescein; GFP: Green fluorescent protein; HATU: Hexafluorophosphate azabenzotriazole tetramethyl uranium; HK: HeLa Kyoto; L-15: Leibovitz's L-15 medium; PBS: Phosphate buffered saline; SEC: Size exclusion chromatography; SOI: Substrate of interest; TCEP: Tris(2-carboxyethyl)phosphine; TEA: Triethylamine; TEOA: Triethanolamine; TFA: Trifluoroacetic acid; THF: Tetrahydrofuran; TIPS: Triisopropylsilane; TMU: Thiol-mediated uptake; Trt: Trityl; WT: Wild type.

2. Synthesis

2.1. Synthesis of CPDs

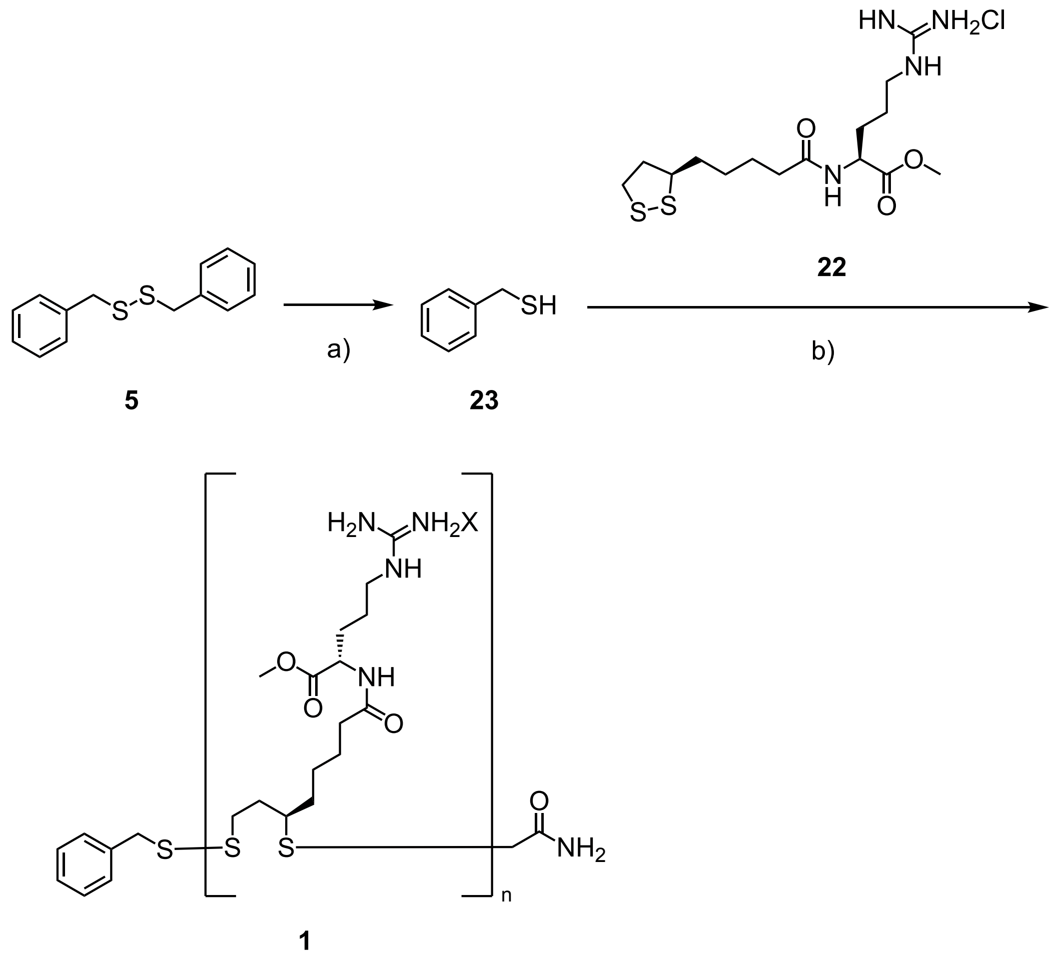

Scheme S1. (a) TCEP, TEOA buffer/DMSO (1:1), 25 °C, 15 min; (b) 1. TEOA buffer/DMF (8:2), 25 °C, 30 min; 2. Iodoacetamide, 25 °C, 15 min. Regioregularity of CPDs not determined, expected are almost randomly mixed 3:2 regio-irregular polymers.

Compound 22 was prepared according to reported procedures.[S2]

Compound 1. The CPDs were synthesized by adapting reported procedures.[S2] Briefly, a stock solution of initiator **23** (I, 25 mM) was prepared by pre-treating a solution of **5** (4 µL of a 25 mM stock solution in DMSO) with TCEP (4 µL of a 25 mM stock solution in 1 M TEOA buffer at pH = 7.0) for 15 min at 25 °C. In the meantime, the monomer **22** (M, 8.3 mg, 20 µmol) was dissolved in DMF (16 µL), followed by 1 M TEOA buffer (pH = 7.0, 76 µL). The pH of

the resulting mixture was adjusted to 7.0, if necessary. To the solution of **22** was added the freshly prepared solution of **23** (8 µL of 25 mM). The polymerization mixture was agitated for 30 min at 25 °C and subsequently quenched with iodoacetamide (25 µL of a 0.8 M stock solution in H_2O). The quenched mixture was stirred for another 10 min, then filtered and purified by GPC according to the absorbance at 255 nm. The collected polymers were lyophilized. The resulting powders were dissolved in 500 µL H_2O and desalted using a Sephadex (PD-MiniTrap G25) column. The obtained solution was lyophilized and dissolved in the desired amount of H_2O to prepare the stock solution. The obtained polymer **1** was characterized using SEC (4.1.). The concentration of the monomer unit (c_M) was determined by the absorbance at 220 nm. The concentration of polymer ($c_P = c_M / n_M$) was estimated from c_M and the number of monomer units/polymer (n_M).

2.2. Synthesis of SOIs

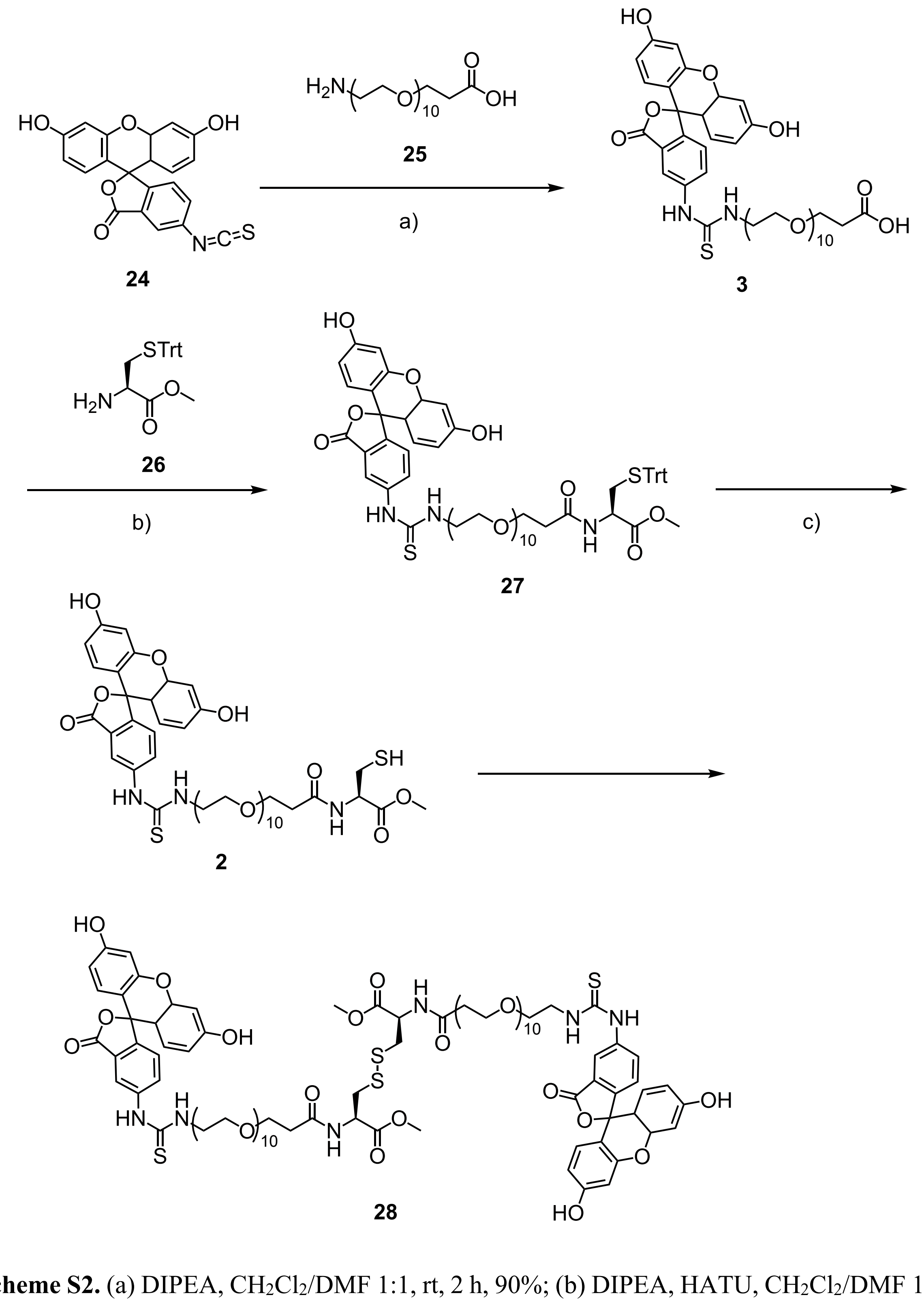

Scheme S2. (a) DIPEA, CH_2Cl_2/DMF 1:1, rt, 2 h, 90%; (b) DIPEA, HATU, CH_2Cl_2/DMF 1:1, rt, 2 h, 72%; (c) TFA, TIPS, CH_2Cl_2, 0 °C to rt, 30 min, 78%.

Compound 3. To a solution of **24** (44 mg, 0.11 mmol) and **25** (40 mg, 75 µmol) in anhydrous CH_2Cl_2/DMF 1:1 (1.2 mL) was added DIPEA (15.8 µL, 90.5 µmol). The reaction mixture was stirred at room temperature for 2 h. The solvent was removed *in vacuo*, and the crude product was directly purified by reverse phase flash chromatography (Scorpius C18e-HP 20 g, 15 mL/min, linear gradient H_2O/CH_3CN + 0.1% TFA from 80:20 to 0:100) to afford **3** as an orange oil (62 mg, 90%). R_f (CH_2Cl_2/MeOH 4:1): 0.27; IR (neat): 2879 (m, C-H), 1722 (m, C=O), 1670 (m, C=O), 1603 (m), 1538 (m), 1454 (m), 1250 (m), 1176 (s, C=S), 1106 (s, C-O), 842 (m, C-H); ^{1}H NMR (500 MHz, DMSO-d_6): 10.10 (br s, 2H), 10.02 (s, 1H), 8.27 (s, 1H), 8.14 – 8.04 (m, 1H), 7.74 (d, $^3J_{H-H}$ = 8.3 Hz, 1H), 7.18 (d, $^3J_{H-H}$ = 8.3 Hz, 1H), 6.67 (d, $^4J_{H-H}$ = 2.3 Hz, 2H), 6.60 (d, $^3J_{H-H}$ = 8.7 Hz, 2H), 6.56 (dd, $^3J_{H-H}$ = 8.7, $^4J_{H-H}$ = 2.3 Hz, 2H), 3.72 – 3.66 (m, 2H), 3.64 – 3.55 (m, 8H), 3.55 – 3.45 (m, 30H), 2.43 (t, $^3J_{H-H}$ = 6.4 Hz, 2H); ^{13}C NMR (126 MHz, DMSO-d_6): 181.0 I, 173.1 (C), 169.0 (C), 160.0 (2×C), 152.4 (2×C), 147.6 (C), 141.8 (C), 129.9 (CH), 129.5 (2×CH), 127.0 (C), 124.6 (CH), 116.8 (CH), 113.1 (2×CH), 110.2 (2×C), 102.7 (2×CH), 83.6 (C), 70.2 (16×CH_2), 70.2 (CH_2), 70.1 (CH_2), 68.9 (CH_2), 66.7 (CH_2), 44.2 (CH_2), 35.2 (CH_2); MS (ESI): 919 ($[M+H]^+$).

Compound 26 was prepared according to reported procedures.[S3]

Compound 27. To a solution of **3** (66 mg, 72 µmol) in a 1:1 mixture of CH_2Cl_2/DMF (2 mL), was added DIPEA (18.7 µL, 107 µmol), then HATU (26 mg, 68 µmol) in DMF (1 mL) followed by a solution of **26** (38 mg, 0.10 mmol) in CH_2Cl_2 (2 mL). The reaction mixture was stirred at room temperature for 2 h. The solvent was evaporated *in vacuo* and the crude product was directly purified by reverse phase flash column chromatography (Scorpius C18e-HP 33 g, 20 mL/min, linear gradient H_2O/CH_3CN + 0.1% TFA from 50:50 to 0:100) to afford **27** as a yellow solid (66 mg, 72%). R_f (CH_2Cl_2/MeOH 9:1): 0.33; Mp: 41 – 42 °C; CD (MeOH): 220 (+3.7); IR (neat): 2872 (m, C-H), 1746 (m, C=O), 1671 (m, C=O), 1593 (m), 1539 (m), 1450 (m), 1384 (m), 1273 (m), 1198 (s), 1173 (s, C=S), 1113 (s, C-O), 847 (m, C-H); ^{1}H NMR (500

MHz, DMSO-d_6): 10.11 (s, 2H), 10.01 (s, 1H), 8.35 (d, $^3J_{H-H}$ = 8.0 Hz, 1H), 8.27 (s, 1H), 8.11 – 8.02 (m, 1H), 7.73 (d, $^3J_{H-H}$ = 8.4 Hz, 1H), 7.37 – 7.22 (m, 15H), 7.17 (d, $^3J_{H-H}$ = 8.4 Hz, 1H), 6.67 (d, $^4J_{H-H}$ = 2.4 Hz, 2H), 6.60 (d, $^3J_{H-H}$ = 8.8 Hz, 2H), 6.56 (dd, $^3J_{H-H}$ = 8.8, $^4J_{H-H}$ = 2.4 Hz, 2H), 4.15 (td, $^3J_{H-H}$ = 8.0, $^3J_{H-H}$ = 5.5 Hz, 1H), 3.74 – 3.64 (m, 2H), 3.63 – 3.42 (m, 45H), 2.41 – 2.29 (m, 2H); ^{13}C NMR (126 MHz, DMSO-d_6): 181.0 (C), 171.2 (C), 170.7 (C), 169.0 (C), 159.9 (2×C), 152.3 (2×C), 147.6 (C), 144.6 (3×C), 141.8 (C), 129.9 (CH), 129.5 (8×CH), 128.6 (6×CH), 127.3 (3×CH), 127.0 (C), 124.5 (CH), 116.8 (CH), 113.0 (2×CH), 110.2 (2×C), 102.7 (2×CH), 83.4 (C), 70.2 (16×CH_2), 70.2 (CH_2), 70.0 (CH_2), 68.9 (CH_2), 67.0 (CH_2), 66.8 (C), 52.5 (CH_3), 51.8 (CH), 42.2 (CH_2), 36.2 (CH_2), 33.3 (CH_2); MS (ESI): 1279 ($[M+H]^+$).

Compound 2. To a solution of **27** (47 mg, 37 µmol) in CH_2Cl_2 (500 µL) was added TFA (500 µL) followed by TIPS (22.8 µL, 111 µmol), at 0 °C. The reaction mixture was allowed to warm to room temperature and was stirred for 30 min. The volatiles were then evaporated *in vacuo* and the crude product was directly purified by reverse phase flash column chromatography (Scorpius C18e-HP 33g, 20 mL/min, linear gradient H_2O/CH_3CN + 0.1% TFA from 95:5 to 0:100) to afford **2** (30 mg, 78%) as an orange oil. R_f (CH_2Cl_2/MeOH 9:1): 0.21; CD (MeOH): 225 (+0.07), 200 (+0.48); IR (neat): 2872 (m, C-H), 1745 (m, C=O), 1671 (m, C=O), 1599 (m), 1538 (m), 1454 (m), 1385 (m), 1275 (m), 1198 (s), 1173 (s, C=S), 1112 (s, C-O), 847 (m, C-H); HRMS (ESI): calcd. for $C_{48}H_{65}N_3O_{18}S_2$ ($[M+Na+H]^{2+}$): 529.6835, found: 529.6814. Compound **2** spontaneously oxidized to compound **28** in DMSO-d_6. ^{1}H NMR (500 MHz, DMSO-d_6): 10.12 (br s, 2H), 10.02 (s, 1H), 8.32 (d, $^3J_{H-H}$ = 7.7 Hz, 1H), 8.27 (s, 1H), 8.12 – 8.04 (m, 1H), 7.74 (d, $^3J_{H-H}$ = 8.4 Hz, 1H), 7.18 (d, $^3J_{H-H}$ = 8.4 Hz, 1H), 6.67 (d, $^4J_{H-H}$ = 2.3 Hz, 2H), 6.60 (d, $^3J_{H-H}$ = 8.7 Hz, 2H), 6.56 (dd, $^3J_{H-H}$ = 8.7, $^4J_{H-H}$ = 2.3 Hz, 2H), 4.46 (td, $^3J_{H-H}$ = 7.7, $^3J_{H-H}$ = 5.1 Hz, 1H), 3.72 – 3.66 (m, 2H), 3.64 (s, 3H), 3.63 – 3.47 (m, 40H), 2.87 – 2.70 (m, 2H), 2.43 – 2.37 (m, 2H); ^{13}C NMR (126 MHz, DMSO-d_6): 181.0 (C), 171.2 (C), 170.8 (C), 169.0 (C), 159.9 (2×C), 152.3 (2×C), 147.6 (C), 141.8 (C), 129.9 (CH), 129.5

(2×CH), 127.0 (C), 124.5 (CH), 116.8 (CH), 113.0 (2×CH), 110.2 (2×C), 102.7 (2×CH), 83.5 (C), 70.2 (16×CH_2), 70.2 (CH_2), 70.0 (CH_2), 68.9 (CH_2), 67.1 (CH_2), 54.9 (CH_3), 52.5 (CH), 44.2 (CH_2), 36.2 (CH_2), 25.9 (CH_2).

Compound 6 was prepared according to reported procedures.[S4]

Compound 9 was prepared according to reported procedures.[S5]

sfGFP S147C Mutant 19 and **mNeonGreen 20** with the following amino acid sequences were produced at the Protein Biochemistry Platform, Faculty of Medicine, University of Geneva, Switzerland.

sfGFP S147C **19**: MSKGEELFTG VVPILVELDG DVNGHKFSVR GEGEGDATNG KLTLKFICTT GKLPVPWPTL VTTLTYGVQC FSRYPDHMKR HDFFKSAMPE GYVQERTISF KDDGTYKTRA EVKFEGDTLV NRIELKGIDF KEDGNILGHK LEYNFNCHNV YITADKQKNG IKANFKIRHN VEDGSVQLAD HYQQNTPIGD GPVLLPDNHY LSTQSVLSKD PNEKRDHMVL LEFVTAAGIT HGMDELYK

mNeonGreen **20**: MVSKGEEDNM ASLPATHELH IFGSINGVDF DMVGQGTGNP NDGYEELNLK STKGDLQFSP WILVPHIGYG FHQYLPYPDG MSPFQAAMVD GSGYQVHRTM QFEDGASLTV NYRYTYEGSH IKGEAQVKGT GFPADGPVMT NSLTAADWCR SKKTYPNDKT IISTFKWSYT TGNGKRYRST ARTTYTFAKP MAANYLKNQP MYVFRKTELK HSKTELNFKE WQKAFTDVMG MDELYK

2.3. Synthesis of TMU Inhibitors

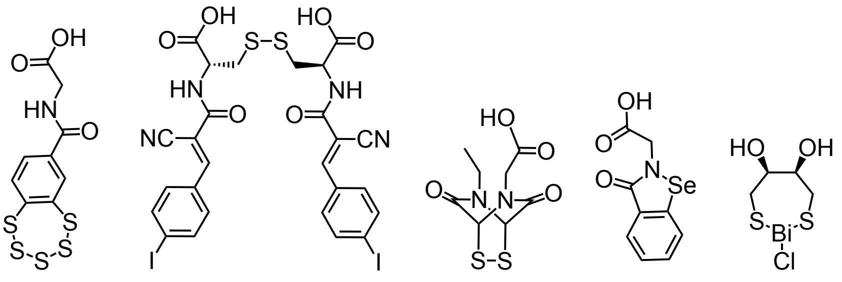

7 (BPS) 8 (dMAC) 29 (ETP) 30 (EBS) 31 (BiC)

Figure S1. Structures of the synthesized TMU inhibitors used throughout this study.

Compounds 7 and **29** were prepared according to reported procedures.[S6]

Compound 8 was prepared according to reported procedures.[S7]

Compound 30 was prepared according to reported procedures.[S8]

Compound 31 was prepared according to reported procedures.[S9]

3. General Procedures for Grafting-to

3.1. Stock Solutions and Buffers Used Throughout the Study

Buffer A:	DPBS, pH 7.0 + 30% CH_3CN	
TCEP	286 g/mol	3 mM in buffer A (if not stated otherwise)
Iodoacetamide	185 g/mol	1 M in ddH_2O
CPDs **1**	-	0.8 – 1.25 mM in ddH_2O
**3**	921 g/mol	50 µM in DMSO
Fl AspA **9**	582 g/mol	3 mM in DMSO
Ac-Cys-OMe **12**	177 g/mol	3 mM in buffer A (if not stated otherwise)
**16**	2725 g/mol	3 mM in CH_3CN/H_2O 2:1
**18**	1029 g/mol	3 mM in CH_3CN/H_2O 2:1
sfGFP S147C **19**	~ 27 kDa	515 µM in PBS (+ 1 mM DTT)

mNeonGreen **20**	~ 27 kDa	653 µM in PBS
**21**	~ 60 kDa	8 µM in ddH_2O
**28**	1056 g/mol	50 µM in DMSO

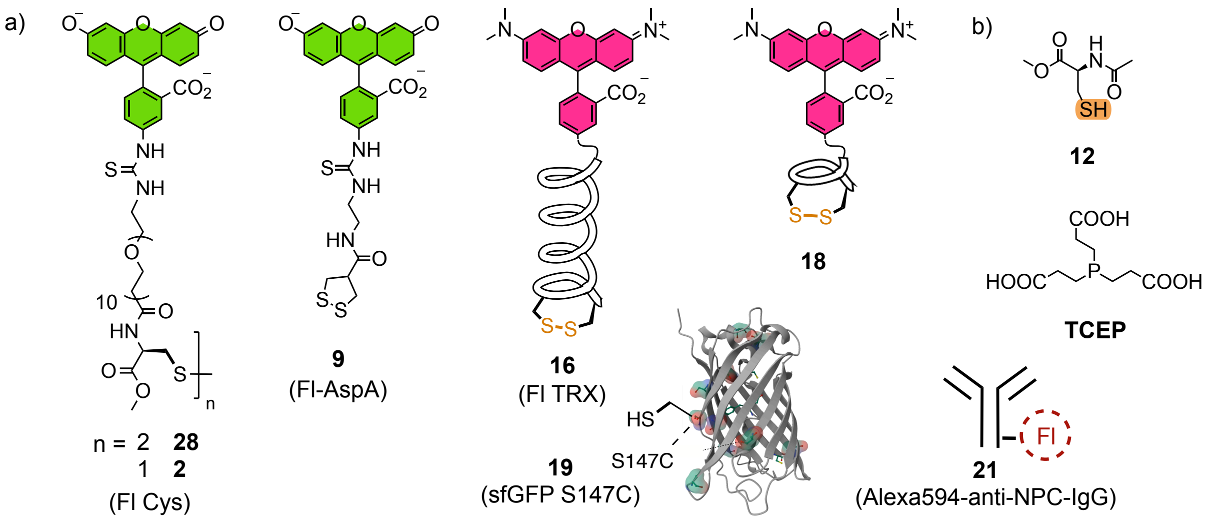

Figure S2. SOIs (a) and reducing agents (b) used in this study.

3.2. General Procedure for Grafting to Small Molecules and IgG

To a solution of the SOI (**28**, **9**, **16**, **18,** or **21**), the desired reducing agent was added (TCEP or Cys **12**, 1 – 2 eq.) to give a mixture of SOI (c_{SOI} = 4 – 1500 µM) and the reducing agent (c = 4 - 1500 µM). The mixture was shaken for 5 min at 25 °C. Afterward, the solution was diluted with buffer A (final c_{SOI} = 1 – 1500 µM), and CPDs **1** (final c_P = 1.3 – 6150 µM; c_P / c_{SOI} = 1.3 – 4.1 eq.) were added. The reaction mixture was shaken at 25 °C for the desired amount of time (5 – 60 min) and then used immediately after preparation. The equivalents of the CPDs **1** are reported for polymer with n_M = 80 and adjusted accordingly when a CPD with a different length was used (*i.e.,* 1.0 equivalent of a polymer with n_M = 80 was considered equivalent to 1.0 × 80/110 = 0.72 equivalent of a polymer with n_M = 110).

3.3. General Procedure for Grafting to sfGFP Mutant 19

The stock solution of **19** was diluted with DPBS to the desired concentration (final concentration 50 nM – 343 µM), and CPDs **1** (final c_P = 65 nM – 1.4 mM; c_P / c_{SOI} = 1.3 – 4.1

eq.) were added. The reaction mixture was shaken at 25 °C for the desired amount of time (1 – 4 h) and used right after preparation. The equivalents of the CPDs **1** are reported for polymer with n_M = 80 and adjusted accordingly when a CPD with a different length was used (*i.e.,* 1.0 equivalent of a polymer with n_M = 80 was considered equivalent to $1.0 \times 80/110 = 0.72$ equivalent of a polymer with n_M = 110).

4. Polymer Characterization by SEC

4.1. Characterization of CPDs

The purified polymer stock solutions (prepared in 2.1.) were diluted with bidistilled H_2O (c_M = 30 µM, 100 µL) and analyzed by SEC, using Superdex 75 10/300 GL with 30% CH_3CN in 0.1 M acetate buffer pH = 6.5 as an eluent at 0.4 mL/min (from 0 to 40 min), then 0.6 mL/min (from 41 to 120 min). UV absorbance was monitored at 220 nm, 255 nm, and 333 nm. The chromatograms obtained at 255 nm (Figure S3a) were used for characterization in comparison with those of molecular weight standards (Figure S3b).

Table S1. Characteristics of compound **1** determined by SEC.[a]

Entry	M_n [kDa][b]	$Đ$[c]	n_M[d]
1	35	1.3	80
2	32	1.3	74
3	41	1.2	94

[a]Polymerization conditions: M = **22**, I = **23**, $[M]_0/[I]_0/[TCEP]_0$ = 100:1:0.5, $[M]_0$ = 200 mM, pH 7.0, 25 °C, 30 min, quenched with iodoacetamide. Polymerizations were repeated multiple times for different batches (entries 1-3). The obtained size and dispersity could vary slightly on batches, but identical trends and properties were observed. Typical batches reported. [b]Number average molecular weight. [c]Dispersity. [d]Theoretical average number of monomers in polymer calculated as $n_M = (M_n - \mathrm{MW[I]} - \mathrm{MW[acetamide]})/\ \mathrm{MW[M]}$.

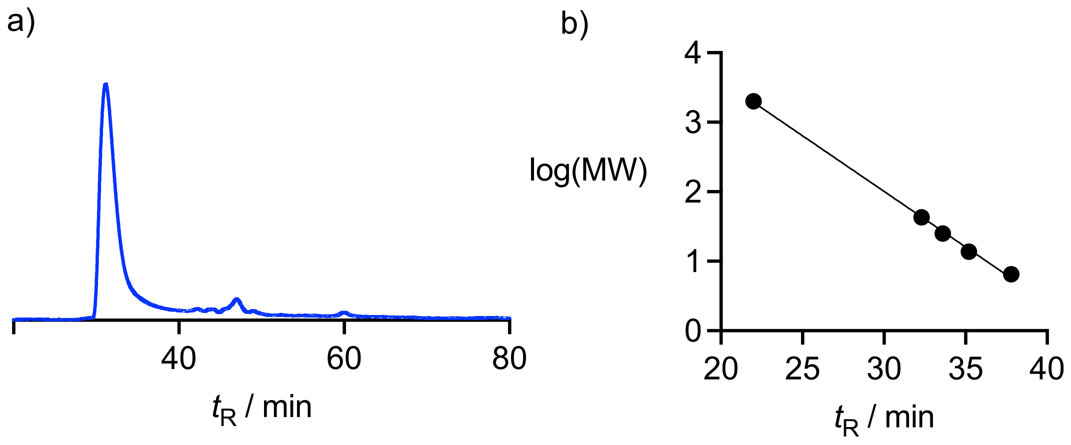

Figure S3. (a) SEC trace of **1**, after purification (λ_{abs} = 255 nm). (b) Calibration of SEC using molecular weight standards (in kDa).

4.2. Characterization of Grafting-to Reaction Mixtures

4.2.1. Grafting CPDs to Fl-Cysteine 2 and 3

The grafting-to solution was prepared according to the *general procedure* (3.2.), treating a 50 µM solution of **28** in DMSO with 2 eq. of TCEP to freshly prepare a 50 µM solution of **2**. Solutions of **2** or **3** were diluted with buffer A to 1 µM for reaction with **1** (4.1 eq. *vs.* SOI, final c_P = 4.1 µM), for 5 min. The mixture was quenched by reacting with iodoacetamide (200 eq. *vs* SOI, final concentration 200 µM) at 25 °C for 5 min. The final solution was filtered and analyzed by SEC (Figure 2c-A, B).

4.2.2. Grafting CPDs to Cyclic Disulfides 9, 16, 18 and Analogues

*SOIs **9**, **16**, **18**.* As in the *general procedure* (3.2.), without the addition of any reducing agent, reacting a 750 µM solution of SOI with **1** (1.3 eq. of an 80 monomers-long polymer, final c_P ~ 1 mM) for 60 min. The mixture was quenched by reacting with iodoacetamide (200 eq., final concentration 150 mM) at 25 °C for 5 min. The final solution was filtered and analyzed by SEC (Figures 2c-D, S4, S5).

*SOIs **13**, **15**, **17**.* As in the *general procedure* (3.2.), the reduced SOIs **13**, **15**, **17** were prepared *in situ* by treating 3 mM stock solutions of the corresponding disulfides (**9**, **16**, **18**,

respectively) with 1 eq. of TCEP (3 mM stock solution in buffer A). The freshly prepared 1.5 mM solutions of **13**, **15**, **17** were diluted with buffer A to 750 µM for reaction with **1** (1.3 eq., c_P ~ 1 mM), for 60 min. The reaction mixture was quenched by addition of 200 eq. of iodoacetamide (final concentration 150 mM), and shaken at 25 °C for 5 min. The final solution was filtered and analyzed by SEC (Figures 2c-C, F, G). When the reaction of **13** with **1** was diluted 75-fold, the SEC chromatograms were comparable with the results obtained for the reaction between **1** and **9** (750 µM of **9**, Figure 2c-D) – in good agreement with cellular experiments (Figure 3a vs. 3c).

*SOIs **9, 16, 18** in the presence of **12**.* The grafting-to solution was prepared according to the *general procedure* (3.2.), using a 3 mM stock solution of SOI, and 1 eq. of **12** (from a 3 mM stock solution in buffer A). Dilution to 750 µM was followed by reaction with **1** (1.3 eq., c_P ~ 1 mM), for 60 min. The reaction mixture was quenched by addition of 200 eq. of iodoacetamide (final concentration 150 mM), and shaking at 25 °C for 5 min. The final solution was filtered and analyzed by SEC (Figures 2c-E, S6, S7).

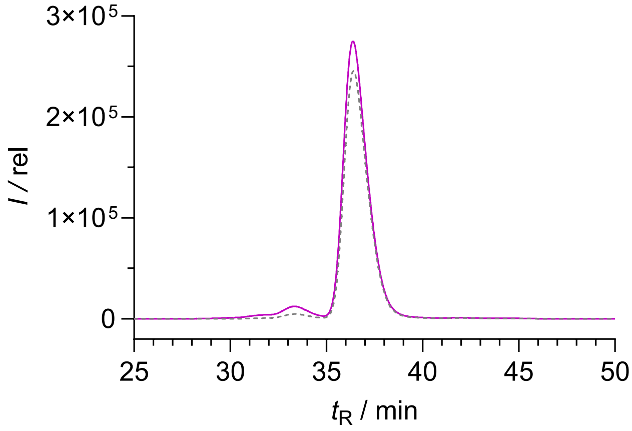

Figure S4. SEC traces of **16** without (dashed) and with (solid) preceding reaction with **1** (1 mM, 750 µM SOI, 60 min).

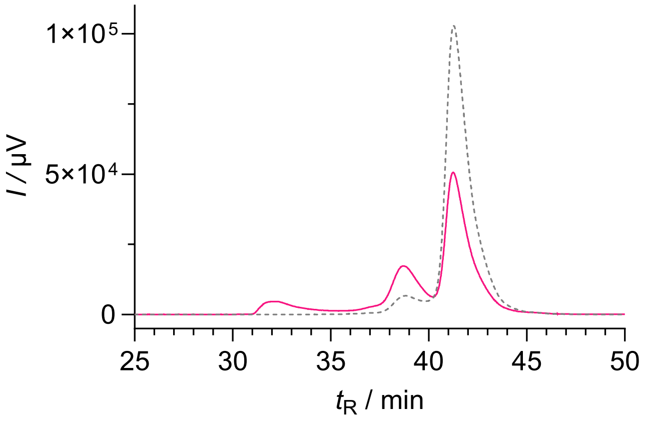

Figure S5. SEC traces of **18** without (dashed) and with (solid) preceding reaction with **1** (1 mM, 750 μM SOI, 60 min).

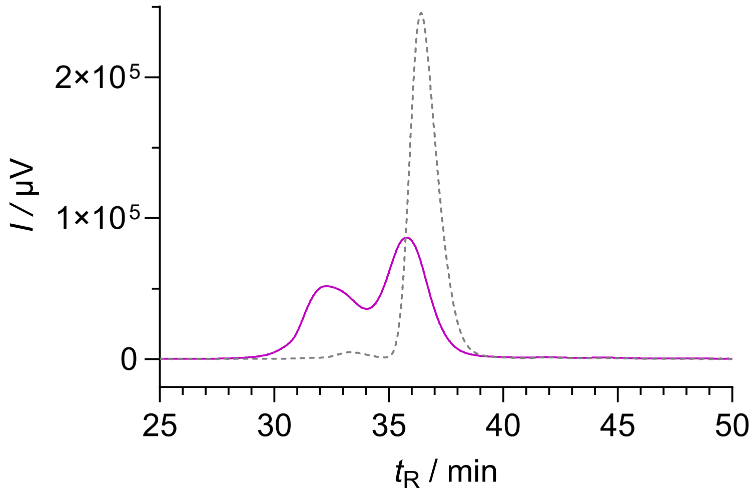

Figure S6. SEC traces of **16** without (dashed) and with (solid) preceding reaction with **1** (1 mM, 750 μM SOI, 60 min) in the presence of **12** (750 μM).

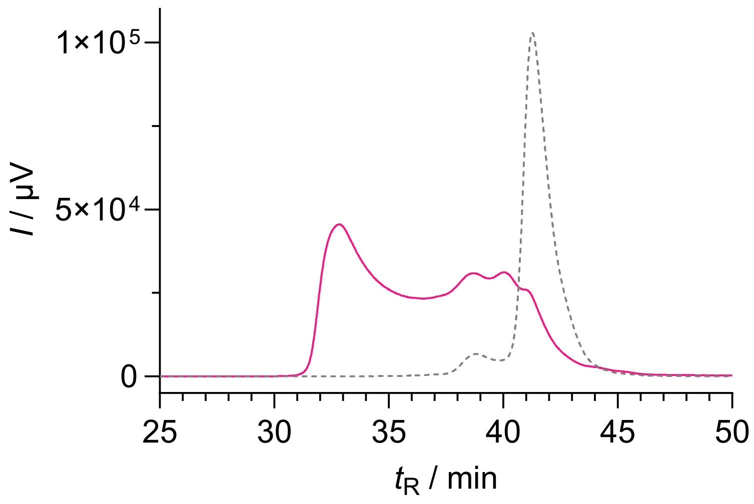

Figure S7. SEC traces of **18** without (dashed) and with (solid) preceding reaction with **1** (1 mM, 750 µM SOI, 60 min) in the presence of **12** (750 µM).

5. Bioconjugation Kinetics

5.1. Preparation of Reaction Mixtures

SOI ***2****.* As in the *general procedure* (3.2.), a 50 µM solution of **28** in DMSO was treated with 2 eq. of TCEP to freshly prepare a 50 µM solution of **2**, which was diluted to 4 µM with buffer A for reaction with **1** (1.3 eq., c_P = 5.2 µM). After the desired time, aliquots of the reaction mixtures were diluted 1:100 and injected into the HPLC for analysis (1 mL/min, from 0 to 2 min: H_2O, from 2 to 30 min: linear gradient H_2O/CH_3CN (+ TFA 0.1%) from 95:5 to 5:95).

SOI ***9****.* As in the *general procedure* (3.2.), using a solution of **9** (final concentration 1.5 mM in buffer A) without addition of any reducing agent, to react with **1** (1.3 eq., c_P = 2.0 mM). After the desired time, aliquots of the reaction mixtures were diluted 1:100 and injected into the HPLC for analysis (1 mL/min, from 0 to 2 min: H_2O, from 2 to 30 min: linear gradient H_2O/CH_3CN (+ TFA 0.1%) from 100:0 to 40:60).

*SOI **13***. As in the *general procedure* (3.2.), using a solution of **9** (3 mM stock solution in DMSO) in the presence of 1 eq. of TCEP (3 mM stock solution in buffer A) to freshly prepare a 1.5 mM solution of **13**, which was reacted with **1** (1.3 eq., c_P = 2.0 mM). After the desired time, aliquots of the reaction mixtures were diluted 1:100 and injected into the HPLC for analysis (1 mL/min, from 0 to 2 min: H_2O, from 2 to 30 min: linear gradient H_2O/CH_3CN (+ TFA 0.1%) from 100:0 to 40:60).

*SOI **15** and **17***. As in the *general procedure* (3.2.), the reduced SOIs **15**, **17** were prepared *in situ* by treating 3 mM stock solutions of the corresponding disulfides in DMSO (**16**, **18**, respectively) with 1 eq. of TCEP (3 mM stock solution in buffer A). The freshly prepared 1.5 mM solutions of **15**, **17** were diluted to 750 µM for reaction with **1** (1.3 eq., c_P ~ 1 mM). After the desired time, aliquots of the reaction mixtures were diluted 1:100 and injected into the HPLC for analysis (1 mL/min, from 0 to 2 min: H_2O, from 2 to 30 min: linear gradient H_2O/CH_3CN (+ TFA 0.1%) from 100:0 to 40:60).

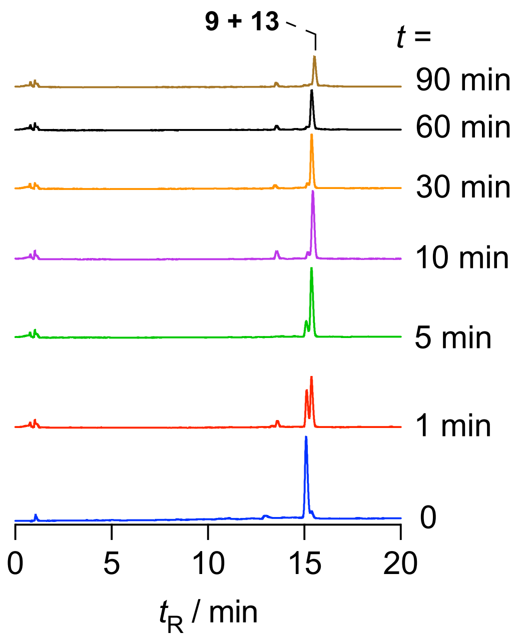

Figure S8. Representative HPLC traces (440 nm) of kinetic studies of reaction of **13** (1.5 mM) with **1** over time.

5.2. Data Analysis

The SOI peak was monitored over time and integrated with an automated protocol on the HPLC software. The areas were normalized to the SOI signal at time t_0 and plotted over time (Figure 2b). Pseudo-first-order and second-order models were used to calculate the rate constants per monomer and per polymer, respectively.

Pseudo-1st Order Kinetics. Since the disulfides (M) in solution were in large excess compared to the SOI, they were considered constant (Equations S1 and S2) according to a pseudo-first order kinetics model (Equation S3). The data were fit to an exponential decay (Equations S4), and the observed rate constant (k_{obs}) was used to calculate the second-order rate constant k_M per disulfides, thus also per monomers (Equation S2). Rate constants obtained from repeated experiments were averaged and reported in Table S2.

$$\frac{\mathrm{d[SOI]}}{\mathrm{dt}} = -k_{\mathrm{M}}[\mathrm{SOI}][\mathrm{M}] \qquad (S1)$$

$$k_{\mathrm{obs}} = k_{\mathrm{M}}[\mathrm{M}] \qquad (S2)$$

$$\frac{\mathrm{d[SOI]}}{\mathrm{dt}} = -k_{\mathrm{M}}[\mathrm{SOI}] \qquad (S3)$$

$$[\mathrm{SOI}] = [\mathrm{SOI}]_0\left(1 - \exp(-k_{obs}\mathrm{t})\right) \qquad (S4)$$

2nd Order Equimolar Kinetics. To obtain a rate constant k per polymer, kinetics were run with about equimolar concentrations of CPD **1** with respect to the SOI (Equation S6). Using this approximation, the kinetics model (Equation S7) becomes solvable and yields a hyperbolic fit (Equation S8). Rate constants obtained from repeated experiments were averaged and reported in Table S2.

$$\frac{\mathrm{d[SOI]}}{\mathrm{dt}} = -k[\mathrm{SOI}][\mathbf{1}] \qquad (S5)$$

$$[\mathrm{SOI}] = [\mathbf{1}] \qquad (S6)$$

$$\frac{\mathrm{d}[\mathrm{SOI}]}{\mathrm{dt}} = -k[\mathrm{SOI}]^2 \qquad (S7)$$

$$[\mathrm{SOI}] = \frac{1}{\frac{1}{[\mathrm{SOI}]_0} + k\mathrm{t}} \qquad (S8)$$

Table S2. Summary of obtained kinetics constants for grafting-to reactions.[a]

Entry	Figure[b]	SOI[c]	k_{obs} [s^{-1}][d]	k_M [M^{-1} s^{-1}][e]	k [M^{-1} s^{-1}][f]
1	2b - A	**2**	$(3 \pm 2)\times10^{-3}$	9 ± 6	1500 ± 900
2	2b - B	**13**	$(2.1 \pm 0.3)\times10^{-4}$	$(2.8 \pm 0.4)\times10^{-3}$	0.26 ± 0.03
3	2b - C	**17**	$(3.5 \pm 0.8)\times10^{-4}$	$(4.7 \pm 0.9)\times10^{-3}$	0.5 ± 0.2
4	2b - C	**15**	$(6 \pm 1)\times10^{-4}$	$(8 \pm 3)\times10^{-3}$	1.0 ± 0.4

[a]Kinetic constants measured according to experimental set-up reported in 5.1. [b]Figures with the corresponding plots. [c]Substrates of interest: reduced **13**, **17, 15** were prepared *in situ* by treating their corresponding disulfides with 1 eq. of TCEP. [d]Pseudo-first-order observed rate constants, obtained by fitting experimental data with Eq. S4. [e]Rate constant per monomer, obtained from Eq. S2. [f]Rate constant per polymer, obtained by fitting the experimental data with Eq. S8.

5.3. Exchange of 2 with Disulfides 4 and 5

To a solution of **2** (5 mM in DPBS/CH_3CN 7:3) freshly prepared by treating **28** with 1 eq. of TCEP, **4** or **5** (2.2 µL of a 20 mM stock solution in DMSO, final concentration 4 mM, final concentration of **2**: 4 mM) were added. The reaction was left shaking at 25 °C. Periodically, the reaction mixture was diluted in 1 M HCl and injected into the RP-HPLC for analysis (1 mL/min, from 0 to 2 min: H_2O, from 2 to 30 min: linear gradient H_2O/CH_3CN (+

TFA 0.1%) from 95:5 to 5:95 – Figure S9a). Data analysis was performed according to the *2nd Order Equimolar Kinetic Model* (5.2.), by fitting the data with Eq. S8 (Figure S9b). Consumption of **2** over time was monitored during the exchange with **4**. However, coelution of **2** and **2-5** made the same approach impracticable during the exchange with dibenzyl disulfide, and **5** was monitored over time in this second case.

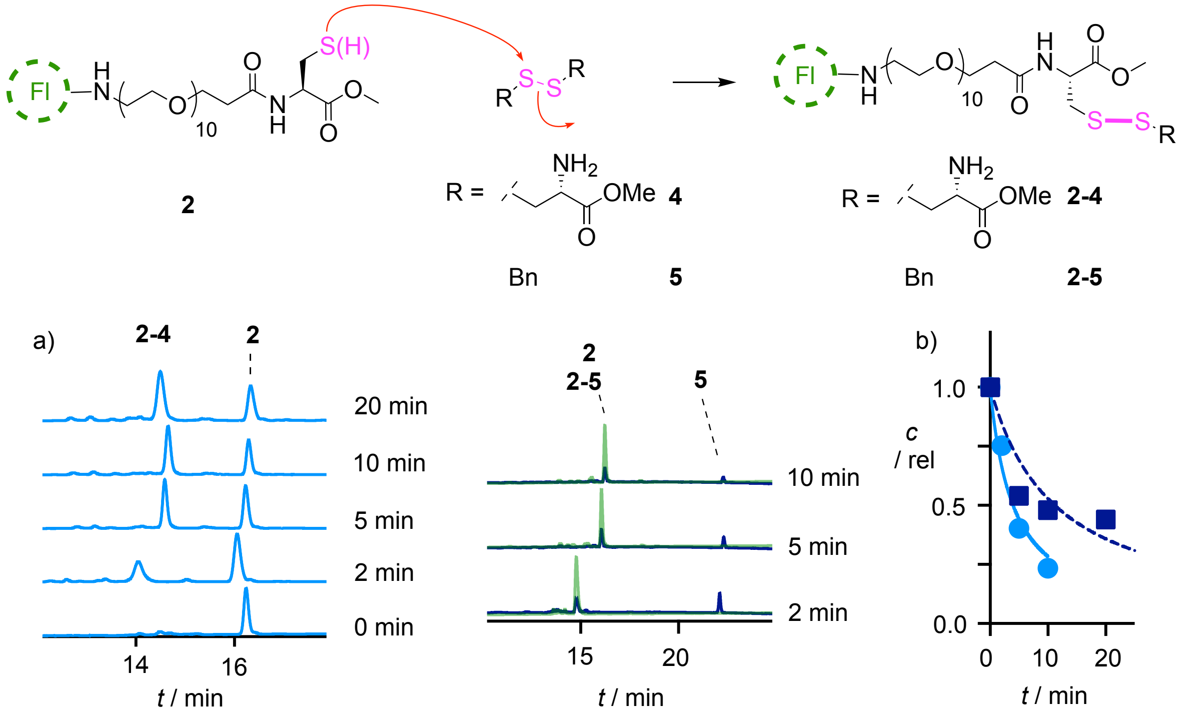

Figure S9. Exchange of **2** (4 mM) with **4** (4 mM) and with **5** (4 mM). RP-HPLC traces of exchange with **4** (440 nm, a) and with **5** (green: 440 nm, dark blue: 205 nm; b). Concentration of starting material over time for exchange of **2** with **4** (squares) and with **5** (circles). The dashed and solid lines are non-linear fit according to Eq. S8, for exchange with **4** and with **5**, respectively.

6. Cell Culture

Human cervical cancer-derived HeLa Kyoto cells were cultured in complete DMEM (GlutaMAX, 4.5 g/L D-glucose), which contains 10% fetal bovine serum (FBS) and 1% Penicillin/Streptomycin (PS). The cells were grown under 5% CO_2 humidified atmosphere at 37 °C on a 75 cm^3 tissue culture flask (TPD Corporation). Cells were harvested by treatment

with 3 mL of phenol-red free TrypLE Express, followed by the addition of 10 mL of complete FDMEM (GlutaMAX, 4.5 g/L D-glucose) at 37 °C. The cells were spun down at 1500 g for 3 min, resuspended in complete FDMEM (GlutaMAX, 4.5 g/L D-glucose), and plated according to the concentration needed.

7. Cellular Uptake

7.1. General Procedure for CLSM Experiments

HeLa Kyoto cells were seeded at 8.0×10^4 cells/mL on a 35 mm glass-bottom dish and cultured overnight. After removing the medium, cells were rinsed with L-15 (3 × 0.8 mL) and treated with the desired fluorescent compound. The cells were incubated at 37 °C under 5% CO_2 atmosphere for 30 min. Then, the medium was removed by aspiration, and cells were rinsed with a 0.1 mg/mL heparin solution in L-15 (3 × 0.8 mL) and kept in L-15 medium for imaging. The distribution of fluorescent compounds was analyzed without fixing by CLSM (Leica SP8), equipped with a 63× oil immersion objective lens. Emission in the green region (Fl, GFP) of the spectrum was measured using λ_{ex} = 488 nm, and emission range from 500 to 550 nm. Red channel was monitored using λ_{ex} = 552 nm, and emission range spanning from 560 to 650 nm.

7.2. Data Analysis

The resulting images were analyzed with FIJI Software. The fluorescence intensity was extracted from the desired channel (green or red), by creating a mask upon application of a threshold on the image and quantified as the mean intensity per pixel.

7.3. Cellular Uptake of Grafted-to Bioconjugates

SOI 2. As in the *general procedure* (3.2.), using DPBS instead of buffer A, a 25 µM stock solution of **28** was treated with 2 eq. of TCEP to freshly prepare a 25 µM solution of **2**, which was diluted to 1 µM (final concentration in DPBS) for reaction with **1** (4.1 eq., c_P = 4.1 µM). After 30 min, the reaction mixture was diluted with fresh L-15 (final **2** concentration 23 nM,

final **1** concentration 94 nM), and added to cells. Incubation of the cells with the prepared mixture, rinsing, and imaging were performed according to the *general procedure* (7.1.).

*SOIs **9**, **16**, **18***. As in the *general procedure* (3.2.), using DPBS instead of buffer A, without the addition of any reducing agent, a 750 μM solution of SOI was treated with **1** (1.3 eq., c_P = ~ 1 mM) for 60 min. Afterward, the reaction mixture was diluted with fresh L-15 (final SOI concentration 75 nM, final **1** concentration 94 nM) and added to cells. Incubation of the cells with the prepared mixture, rinsing, and imaging were performed according to the *general procedure* (7.1.).

*SOIs **13, 15, 17***. As in the *general procedure* (3.2.), using DPBS instead of buffer A, the reduced SOIs **13**, **15**, **17** were prepared *in situ* by treating 3 mM stock solutions of the corresponding disulfides (**9**, **16**, **18**, respectively) with 1 eq. of TCEP (3 mM stock solution in DPBS). The freshly prepared 1.5 mM solutions of **13**, **15**, **17** were diluted to 750 μM (final concentration, in DPBS) for reaction with **1** (1.3 eq., c_P ~ 1 mM), for 60 min. Afterward, the reaction mixture was diluted with fresh L-15 (final SOI concentration 75 nM, final **1** concentration 94 nM), and added to cells. Incubation of the cells with the prepared mixture, rinsing, and imaging were performed according to the *general procedure* (7.1.).

*SOIs **9**, **16**, **18** in the presence of **12***. The grafting-to solution was prepared according to the *general procedure* (3.2.), using DPBS instead of buffer A, a 3 mM stock solution of SOI, and 1 eq. of **12** (from a 3 mM stock solution in DPBS). Dilution to 750 μM was followed by reaction with **1** (1.3 eq., c_P ~ 1 mM), for 60 min. Afterward, the reaction mixture was diluted with fresh L-15 (final SOI concentration 75 nM, final **1** concentration 94 nM), and added to cells. Incubation of the cells with the prepared mixture, rinsing, and imaging were performed according to the *general procedure* (7.1.).

*SOI **19***. As in the *general procedure* (3.3.), **19** (50, 100, 500, 1000 nM in DPBS) was treated with **1** (4.1 eq., c_P = 0.21, 0.41, 2.0, 4.1 μM, respectively) for the desired time (1 h or 4

h). Afterward, the reaction mixture was diluted with fresh L-15 (final **19** concentration 23 nM, final **1** concentration 94 nM), and added to cells. Incubation of the cells with the prepared mixture, rinsing, and imaging were performed according to the *general procedure* (7.1.).

*SOI **21***. As in the *general procedure* (3.2.), using DPBS instead of buffer A, and treating **21** (8 μM stock solution in ddH_2O) with 1 eq. of TCEP (8 μM stock solution in DPBS), before addition of **1** (4.1 eq., c_P = 16.4 μM; final **21** concentration = 4 μM). After 4 h, the reaction mixture was diluted with L-15 (final **21** concentration 23 nM, final **1** concentration 94 nM), and added to cells. Incubation of the cells with the prepared mixture, rinsing, and imaging were performed according to the *general procedure* (7.1.).

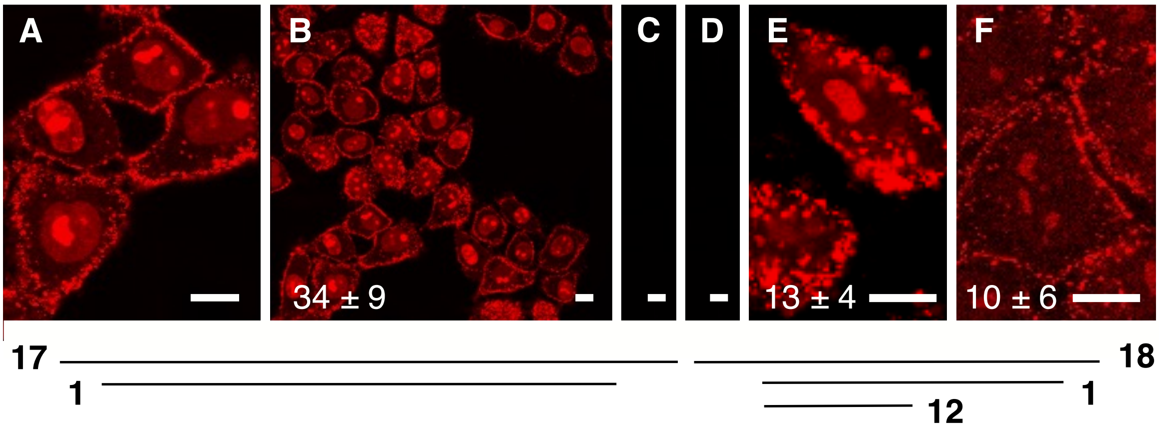

Figure S10. CLSM images of HK cells upon incubation with **17** (A-C), and **18** (D-F, c_u = 75 nM, t_{bc} = 30 min), without (C, D) and after bioconjugation with **1** (A-B, E-F; c_{bc} = 750 μM, 1.25 eq. **1**, t_{bc} = 1 h), without (A-D, F) or with **12** (1 eq., E). Non-comparable image intensities; intensity changes ±**1**: I / I_0 ± SD (B, E, F, I_0: C, D); scale bars: 10 μm.

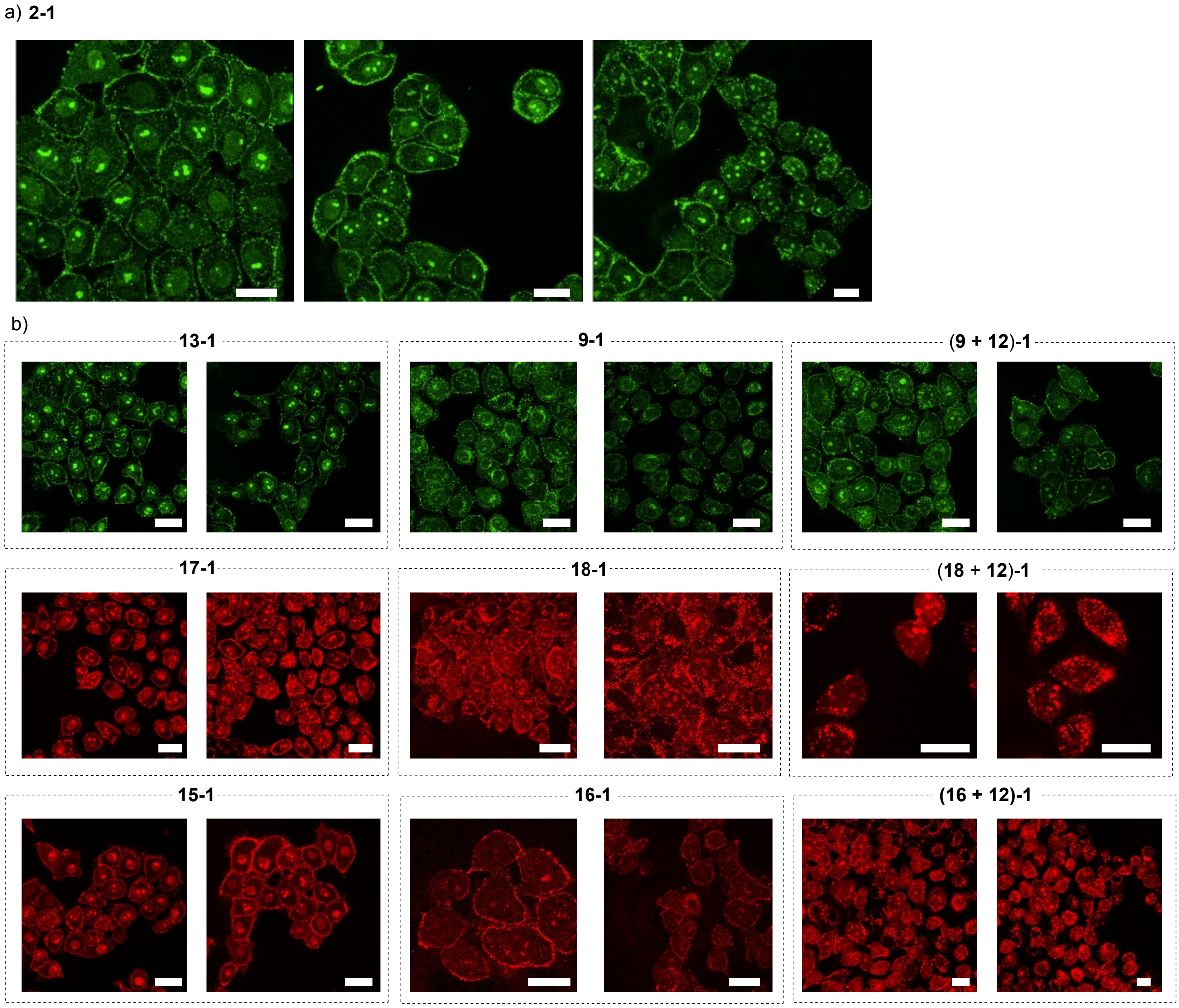

Figure S11. CLSM images of HK cells incubated with indicated SOIs (a: 23 nM, b: 75 nM, L-15, 30 min) prepared by reaction of SOIs with **1** as reported in 7.3. Scale bars: 20 μm. These images are (some of the) technical replicates of conditions already reported in the main manuscript (Figures 2, 3). Intensities are not comparable, averaged increase ± **1** is reported in the manuscript.

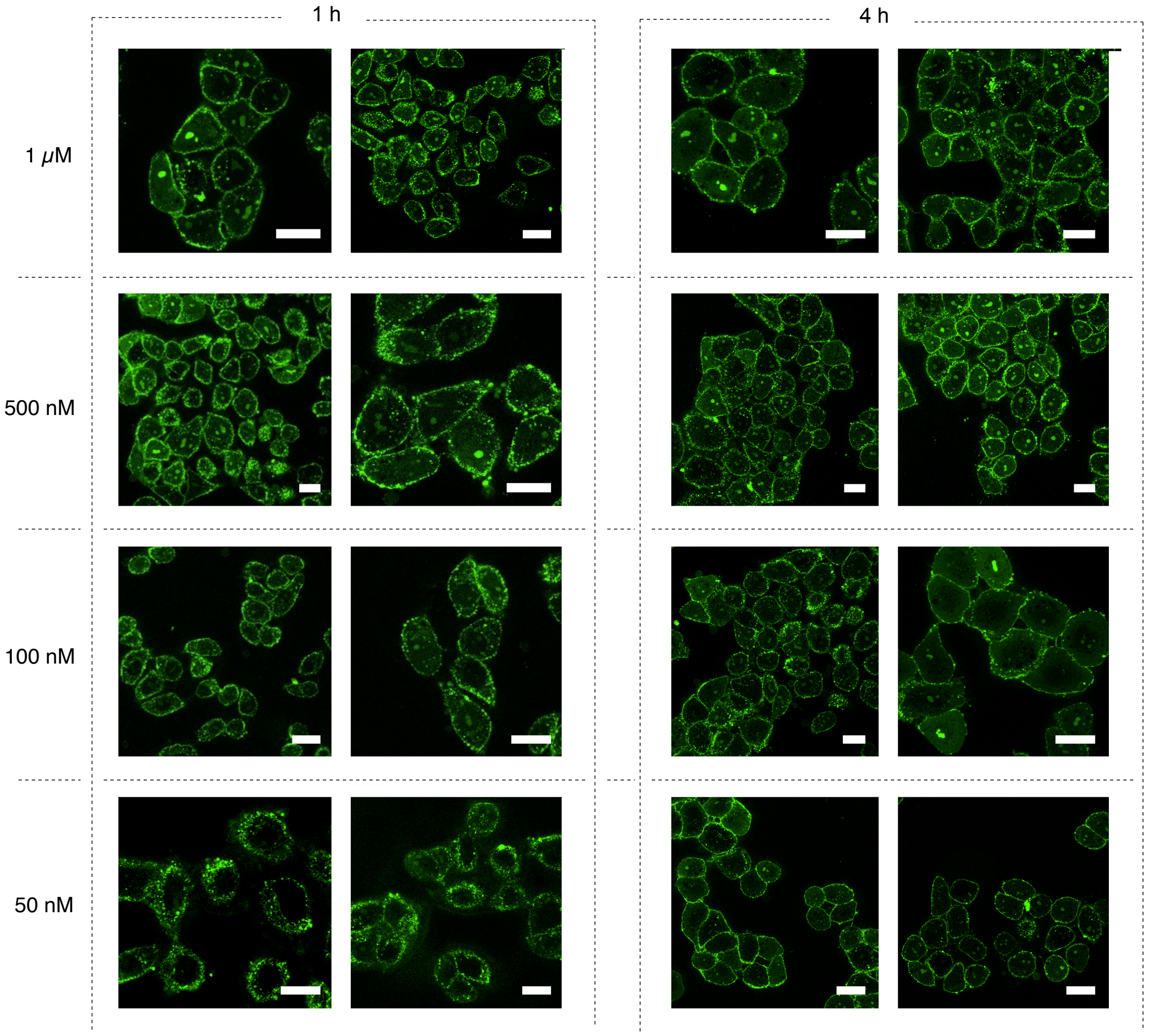

Figure S12. CLSM images of HK cells incubated with **19-1** (23 nM, L-15, 30 min) prepared by reaction of **19** (1000-500-100-50 nM top to bottom, 1 h (left) or 4 h (right)) with **1** (4.1 eq.) as reported in 7.3. Scale bars: 20 μm. These images are (some of the) technical replicates of conditions already reported in the main manuscript (Figure 4). Intensities are not comparable, average increase ± **1** is reported in the manuscript.

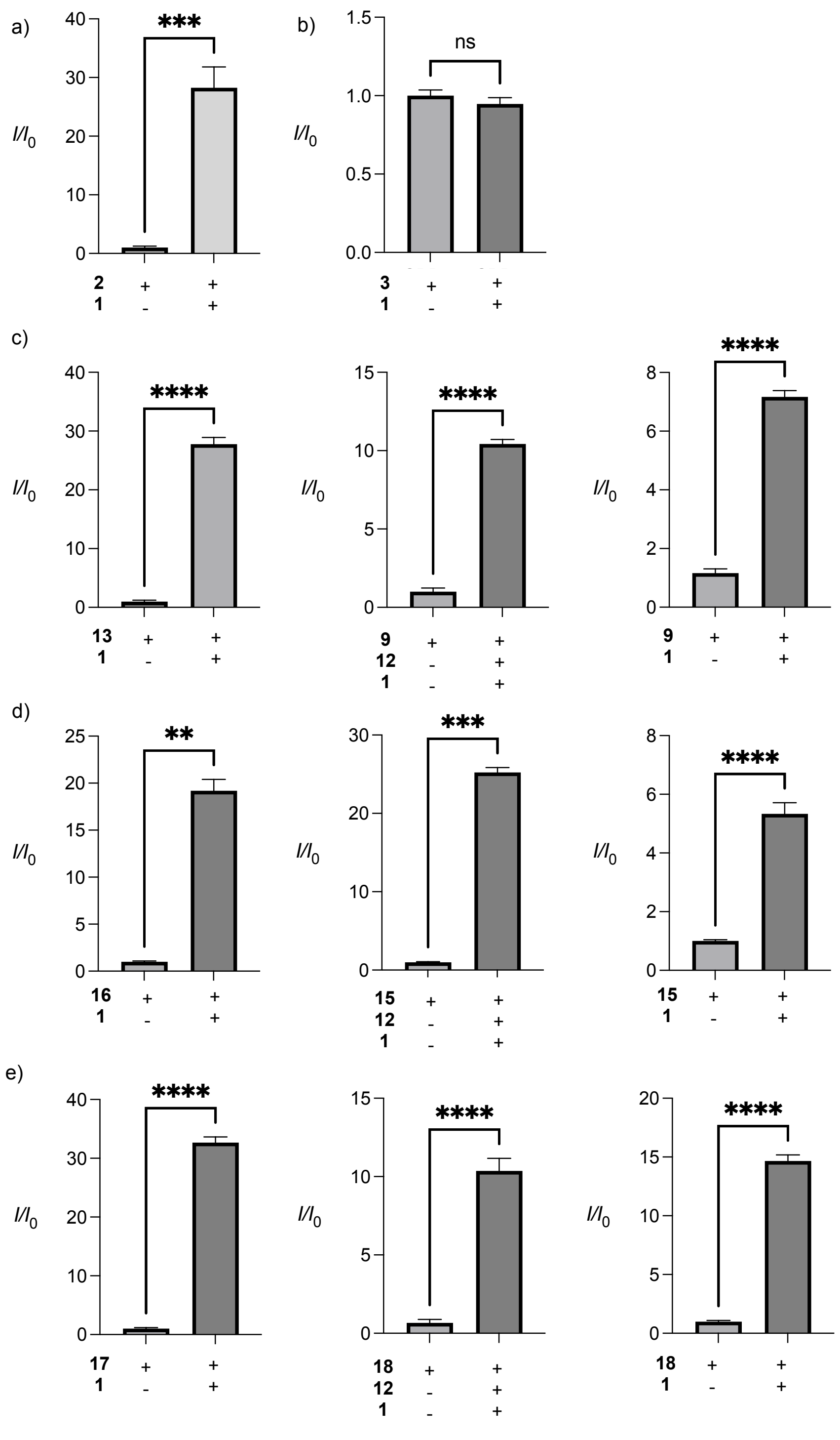

Figure S13. Intensity enhancement measured by CLSM in HK cells, upon incubation of HK cells of SOIs **2** (a), **3** (b), **9**, **13** (c), **15**, **16** (d), **17**, **18** (e) without and after reaction with **1**, according to procedures described in 7.3, with the results of nonparametric two-tailed *t*-tests ($P > 0.1234$: ns, < 0.0021: **, < 0.0002: ***, < 0.0001: ****). Error bars represent SEM.

7.4. Cell Viability Assay

Cell viability after treatment with grafted-to **1** was evaluated using Cell Titer Blue (Promega, G8080). Briefly, cells were seeded in a 96-well plate at 12 000 cells/well in complete FDMEM and left incubating under 5% CO_2 humidified atmosphere at 37 °C overnight. Afterward, the medium was removed, and cells were washed with fresh L-15 (3 × 0.2 mL), ending the washing procedure with 100 μL of L-15 in each well. Then, the desired SOI-**1** conjugate (50 μL of a 3× final concentration stock solution in L-15) was added. After 30 min incubation at 37 °C under 5% CO_2 atmosphere, cells were rinsed with fresh L-15 (3 × 0.2 mL), ending the washing step with 100 μL of L-15 in each well. Cell-Titer Blue dye was added (20 μL/well) to the cells, and the cells were incubated at 37 °C under 5% CO_2 atmosphere for 4 h. Afterwards, the plate was analyzed by fluorescence spectroscopy, using a MicroWell plate reader MicroMax 384 coupled to a fluorometer (λ_{ex} = 530 nm, λ_{em} = 580 nm, slits em/ex 10/10 nm). Intensity values from wells with untreated cells were set as 100% viability. Positive controls were prepared by treating cells with different concentrations of poly[R] (40 μM and 100 μM in L-15, final concentrations). SOI-**1** conjugates, **2-1**, **9-1, 19-1,** were prepared as in 7.3., while **16-1** and **18-1** were not tested since Cell-Titer Blue assay is not compatible with red-emitting fluorophores (Figure S14a).

In a different experiment, three different batches of CPDs **1** (of considerably different length, from n_M = 32 to 110) were tested at different concentrations for viability. Monomer concentration c_M was varied between 12.5 μM and 25 μM (Figure S14b). Despite a slight length-dependent toxicity, working below 10 μM monomer concentration (*i.e,* 125 nM of an 80 monomers-long polymer) prevents a drop in the cellular viability with all the tested CPDs.

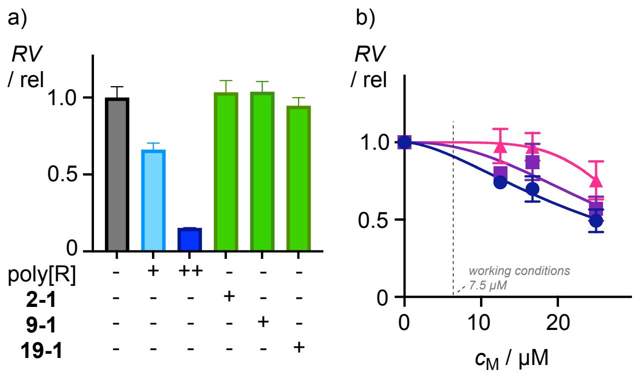

Figure S14. Viability of HK cells after treatment with (a) L-15, poly[R] (40 µM and 100 µM monomer in L-15), **2-1**, **9-1**, **19-1** (23 nM of SOI in L-15 prepared as described in 7.1), and with (b) different monomer concentrations (in L-15) of **1** with n_M = 32 (magenta), 47 (purple), 110 (blue). Error bars represent SEM from technical triplicates.

7.5. Colocalization Experiments

7.5.1. Colocalization of 9-1 with Golgi Tracker 32

Sample preparation. As in the *general procedure* (3.2.), using DPBS instead of buffer A, the reduced **13** was prepared *in situ* by treating 3 mM stock solution of **9** with 1 eq. of TCEP (3 mM stock solution in DPBS). The freshly prepared 1.5 mM solution of **13** was diluted to 10 µM (final concentration, in DPBS) for reaction with **1** (4.1 eq., c_P ~ 41 µM) for 60 min. Afterward, the reaction mixture was diluted with fresh L-15 (final **13** concentration 23 nM, final **1** concentration 94 nM) and used immediately.

Cell Experiment. The HK cells were incubated with the mixture prepared above (7.5.1.), at 37 °C under 5% CO_2 atmosphere for 30 min. Then, the medium was removed by aspiration, and the cells were rinsed with a 0.1 mg/mL heparin solution in L-15 (3 × 0.8 mL). Then, SiR-AspA **32**[S10] (500 nM solution in L-15) was added and the cells were incubated for further 10 min. Lastly, the medium was removed by aspiration, and the cells were rinsed with L-15 (3 × 0.8 mL), and kept in fresh L-15 for imaging. The distribution of fluorescent compound was

analyzed as in the *general procedure* (7.1.), acquiring simultaneously the green channel (FITC, λ_{ex} = 488 nm, λ_{em} = 500-575 nm) and the deep-red channel (SiR, λ_{ex} = 638 nm, λ_{em} = 650-740 nm).

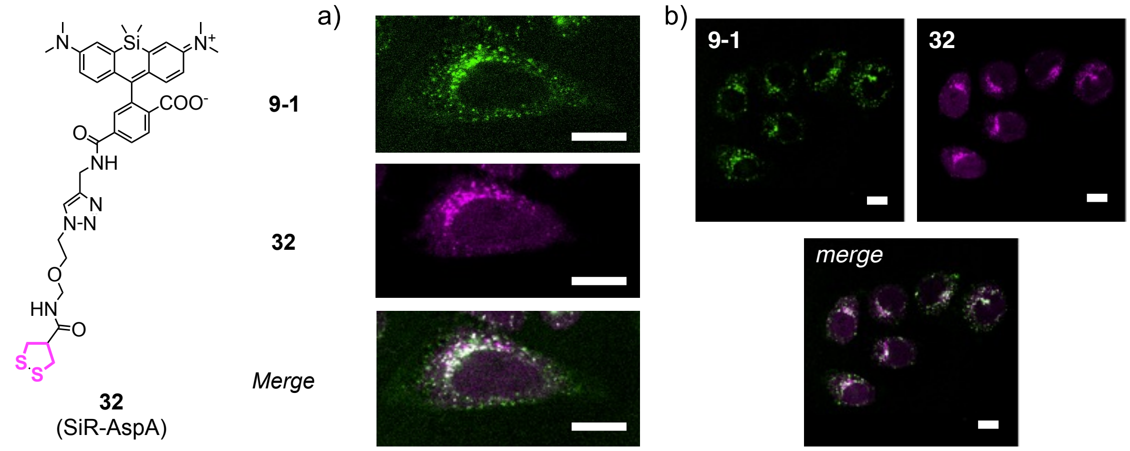

Figure S15. Co-localization experiment of **9-1** with Golgi Tracker **32**. CLSM images (more (a) and less (b) zoomed) of HK cells incubated with *i)* **9-1** (23 nM, L-15, 30 min) after reaction of **9** (10 µM) with **1** (4.1 eq., 60 min, DPBS), and then *ii)* **32** (500 nM, L-15, 10 min). Scale bars: 10 µm. Manders coefficient = 0.64, determined by ImageJ “coloc 2” plugin.

7.5.2. Nucleoli Staining

Nucleolar localization of CPD conjugates was evidenced by colocalization of the bright spots in CLSM images with the dark compartments in brightfield images.[S11]

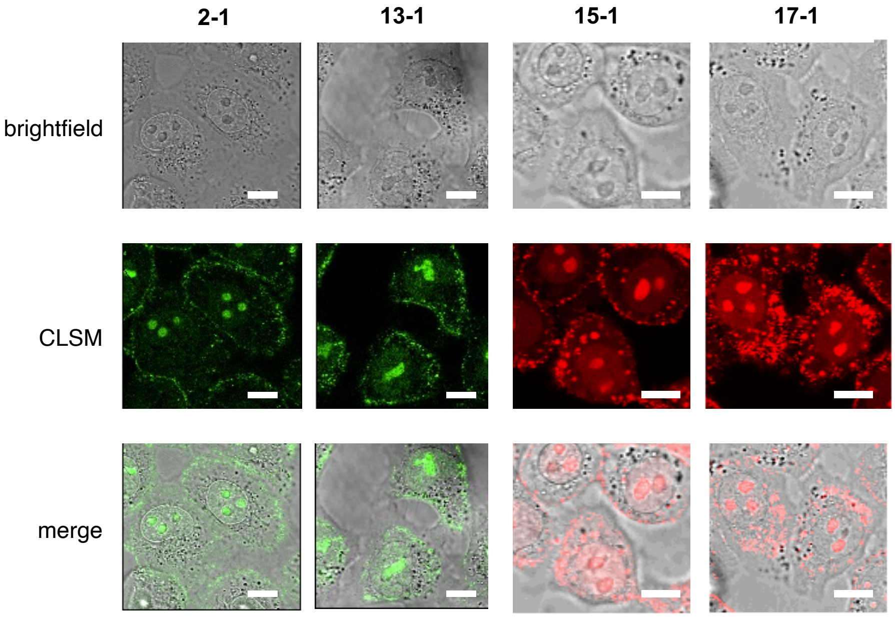

Figure S16. Brightfield (top), confocal (middle) images and their merge (bottom) of HK cells incubated with conjugates **2-1** (23 nM, L-15, 30 min), **13-1**, **15-1**, **17-1** (75 nM, L-15, 30 min) after reaction of their corresponding SOIs (1 µM for **2**, 750 µM for **13**, **15**, **17**) with **1** (4.1 eq., 30 min, DPBS for **2**; 1.25 eq., 60 min, DPBS for the others). Scale bars: 10 µm.

8. Inhibition Assay

8.1. General Procedure

HeLa Kyoto cells were seeded at 8.0 × 10^4 cells/mL on a 35 mm glass-bottom dish and cultured overnight. After removing the medium, cells were rinsed with L-15 medium (3 × 0.8 mL) and treated with 0.9 mL of inhibitor solution in L-15 medium. The cells were incubated at 37 °C under 5% CO_2 atmosphere for 1 h. Afterward, the SOI-**1** conjugate (100 µL of a 10x solution in L-15, prepared as in 7.3.) was added, and cells were incubated for further 20 min at 37 °C under 5% CO_2 atmosphere. Lastly, the medium was removed by aspiration, and cells were rinsed with fresh L-15 medium (3 × 0.3 mL) and kept in L-15 medium for imaging. The

distribution of fluorescent compounds was analyzed without fixing by CLSM (Leica SP8), equipped with a 63× oil immersion objective lens. Emission in the green region (Fl, GFP) of the spectrum was measured using λ_{ex} = 488 nm, and emission range from 500-550 nm. Red channel was monitored with λ_{ex} = 552 nm, and emission range spanning from 560 to 650 nm. Intensity from cell images was extracted as in 7.2.

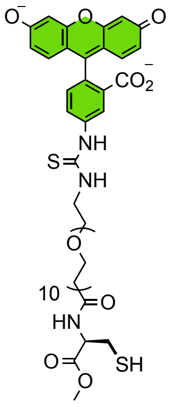

2

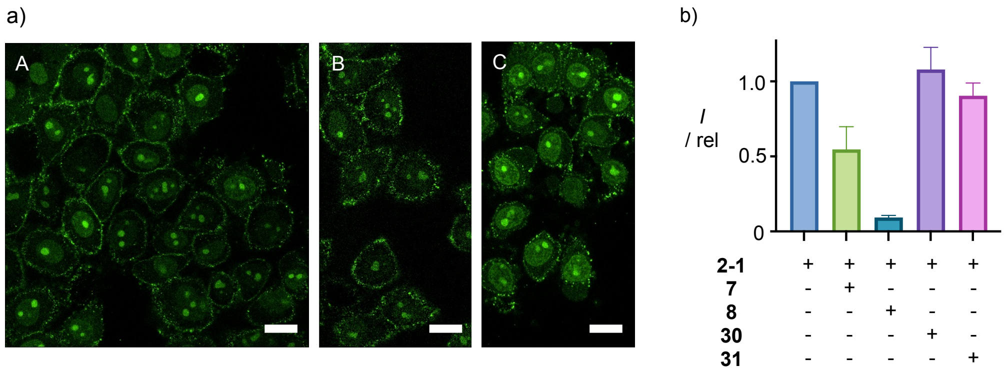

Figure S17. CLSM images (a) of HK cells treated with **2-1** (23 nM, L-15, 20 min, A) prepared by reaction of **2** (1 μM) with **1** (4.1 μM, DPBS, 30 min), after preincubation with TMU inhibitors **30** (50 μM, B) and **31** (3 μM, C). Corresponding quantification is reported in (b), including data from Figures 2d, C-E. Error bars represent SEM from triplicate. Scale bars: 20 μm.

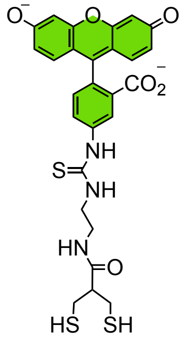

13

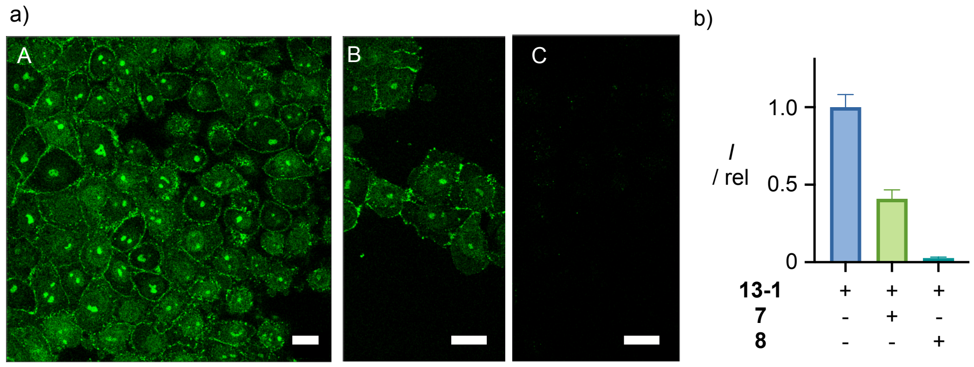

Figure S18. CLSM images (a) of HK cells treated with **13-1** (75 nM, L-15, 20 min, A) prepared by reaction of **13** (750 μM) with **1** (940 μM, DPBS, 30 min), after preincubation with TMU

inhibitors **7** (5 µM, B) and **8** (50 µM, C). Corresponding quantification is reported in (b). Error bars represent SEM. Scale bars: 20 µm.

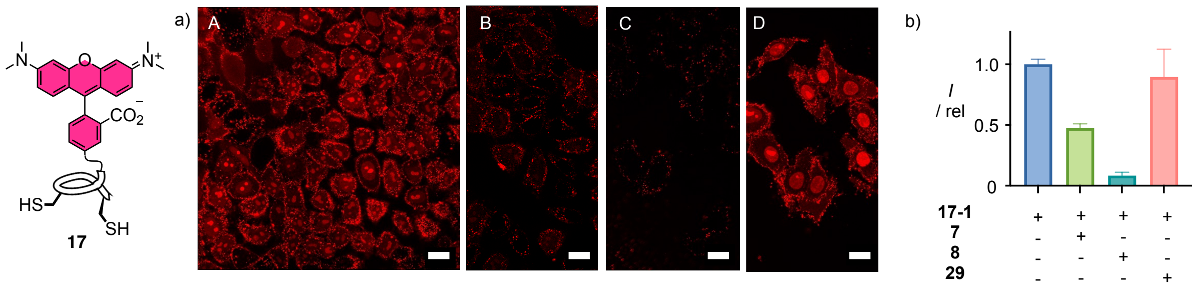

Figure S19. CLSM images (a) of HK cells treated with **17-1** (75 nM, L-15, 20 min, A) prepared by reaction of **17** (750 µM) with **1** (940 µM, DPBS, 30 min), after preincubation with TMU inhibitors **7** (5 µM, B), **8** (50 µM, C), and **29** (30 µM, D). Corresponding quantification is reported in (b). Error bars represent SEM. Scale bars: 20 µm.

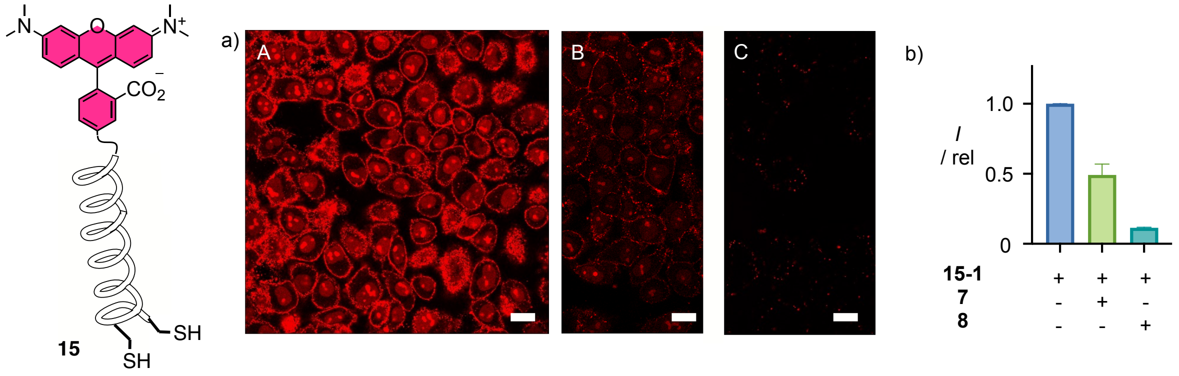

Figure S20. CLSM images (a) of HK cells treated with **15-1** (75 nM, L-15, 20 min, A) prepared by reaction of **15** (750 µM) with **1** (940 µM, DPBS, 30 min), and preincubating the cells with TMU inhibitors **7** (5 µM, B), **8** (50 µM, C). Corresponding quantification is reported in (b). Error bars represent SEM. Scale bars: 20 µm.

9. Measurement of Reduction Potentials

9.1. General Procedure

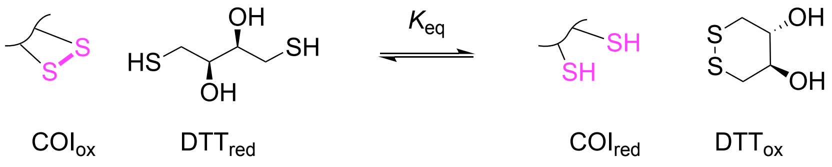

Scheme S3. Reduction potential measurement by reduction of a compound of interest (COI) upon treatment with equimolar amount of DTT.

The following protocol was prepared adapting reported procedures.[S12, S13] To a solution of disulfide COI in 0.1 M phosphate buffer (150 µL, 100 µM), an equimolar amount of DTT was added (1.5 µL of a 10 mM stock solution in 0.1 M phosphate buffer, final concentration 100 µM). The reaction was left at 25 °C under N_2 atmosphere. Regularly, 40 µL of the reaction mixture were quenched with 10 µL of HCl 1 M (final disulfide concentration 80 µM) and analyzed by HPLC. The evolution of oxidized and reduced forms of COI was monitored over time, and their peak integrals were compared to estimate [COI$_{ox}$] and [COI$_{red}$]. The equilibrium was considered reached when the results were stable for three consecutive time points.

The equilibrium constant K_{eq} of the reaction mixture was calculated as follows. Generic equation S9 could be converted into S11, since equimolar amounts of DTT and COI were used (Equation S10). Then, the K_{eq} was determined using [COI$_{ox}$] and [COI$_{red}$] at equilibrium and converted into the reduction potential by using Nernst Equation S12, where E^0_{DTT} = -327 mV,[S12, S14] n = 2, R = 8.314 J mol^{-1} K^{-1}, F = 96485 C mol^{-1}, and T is the temperature expressed in K.

$$K_{eq} = \frac{[\mathrm{COI_{red}}][\mathrm{DTT_{ox}}]}{[\mathrm{COI_{ox}}][\mathrm{DTT_{red}}]} \qquad \text{(S9)}$$

$$[\mathrm{COI_{ox}}] = [\mathrm{DTT_{red}}], \text{and } [\mathrm{COI_{red}}] = [\mathrm{DTT_{ox}}] \qquad \text{(S10)}$$

$$K_{eq} = \frac{[\mathrm{COI_{red}}]^2}{[\mathrm{COI_{ox}}]^2} \tag{S11}$$

$$E^0_{\mathrm{COI}} = E^0_{\mathrm{DTT}} - \frac{RT}{nF}\ln\frac{1}{K_{eq}} \tag{S12}$$

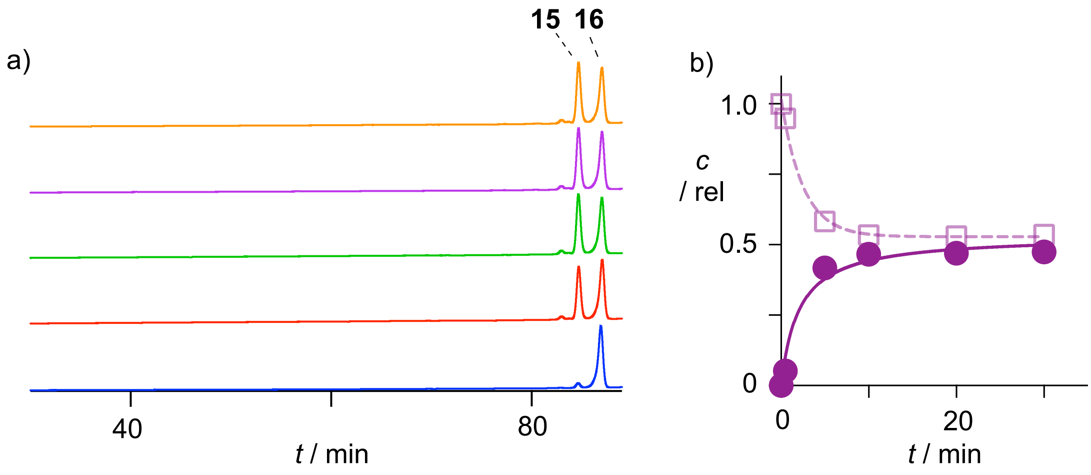

Figure S21. Reduction potential measurement of **16** at 25 °C. (a) HPLC traces (1 mL/min, from 0 to 15 min: H_2O, from 15 to 90 min: linear gradient H_2O/CH_3CN (+ TFA 0.1%) from 100:0 to 70:30; monitored at 550 nm) of the reaction mixture at t = 0.1, 5, 10, 20, 25 min (from bottom to top). (b) Relative concentration of **15** (filled circles) and **16** (empty squares) over time.

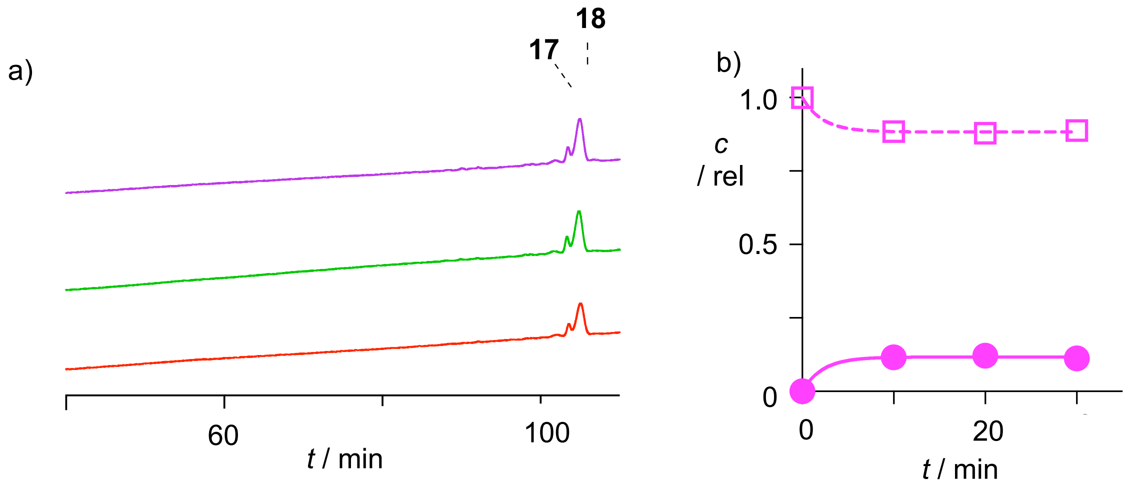

Figure S22. Reduction potential measurement of **18** at 25 °C. (a) HPLC traces (1 mL/min, from 0 to 15 min: H_2O, from 15 to 145 min: linear gradient H_2O/CH_3CN (+ TFA 0.1%) from 100:0 to 70:30; monitored at 550 nm) of the reaction mixture at t = 10, 20, 25 min (from bottom to top). (b) Relative concentration of **17** (filled circles) and **18** (empty squares) over time.

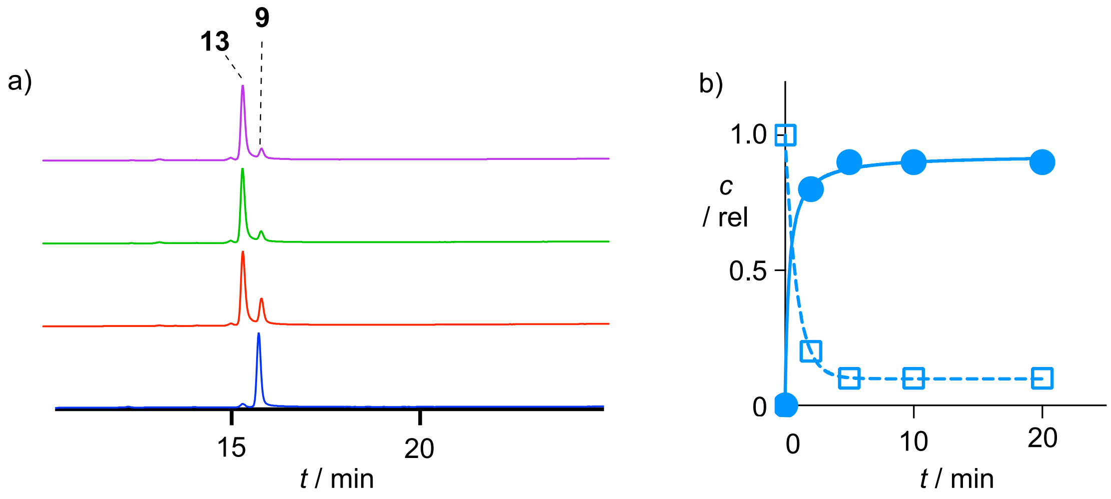

Figure S23. Reduction potential measurement of **9** at 25 °C. (a) HPLC traces (1 mL/min, from linear gradient H_2O/CH_3CN (+FA 0.1%) from 95:5 to 70:30; monitored at 254 nm) of the reaction mixture at t = 2, 5, 10, 20 min (from bottom to top). (b) Relative concentration of **13** (filled circles) and **9** (empty squares) over time.

9.2. Effect of Helix Secondary Structure on the Redox Potential

To evaluate the effect of the secondary structure on the properties of the disulfide **16**, the reduction potential was also determined for thermally denatured peptide. CD spectrum of a non-fluorescent analog of **16** (namely, **33** – Figure S24) was measured at different temperatures, to evaluate the thermal denaturation of its secondary structure. To do so, CD spectrum of a 79 µM solution of **33** in 10 mM phosphate buffer was measured between 10 and 60 °C (Figure S24a). After this heating ramp, cooling down the system to 20 °C showed recovered helicity, proving reversibility of the thermal unfolding (Figure S24b).

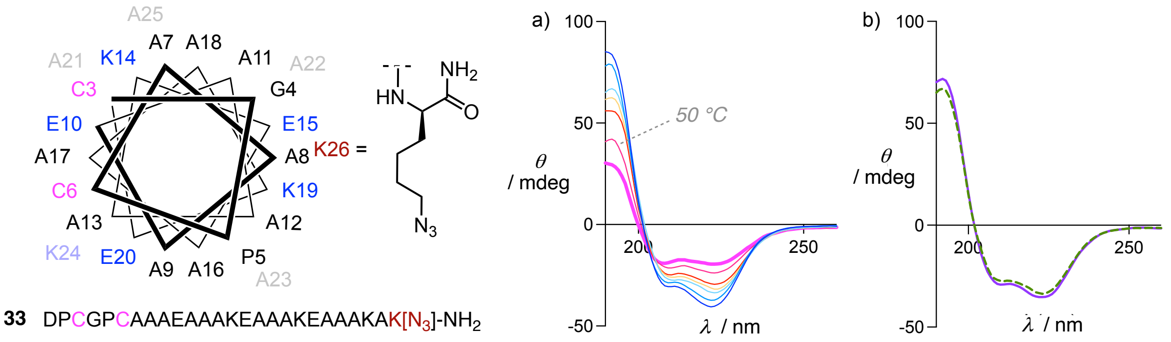

Figure S24. Non-fluorescent analogue **33** of TRX **16**. (a) CD spectrum of **33** (79 µM in 10 mM phosphate buffer – pH = 7.3), at different temperatures: 10, 15, 17, 20, 25, 30, 40, 50, 60 °C (from blue to magenta). (b) CD spectrum of **33** (79 µM in 10 mM phosphate buffer – pH = 7.3) at 17 °C before (dashed green line) and after (solid purple line) heating cycle (10-70 °C).

CD spectroscopy showed that the secondary structure of TRX mimics was unfolded at 50 °C. Thus, aiming at studying the effect of the secondary structure on the disulfide reduction potential at the N terminus of **16**, the reduction potential of **16** was measured at 50 °C (Figure S25). The reaction and data analysis were performed as in the *general procedure* (9.1.), preincubating the COI at 50 °C for 10 minutes, to ensure full denaturation. For comparison, the reduction potential of **18** was also measured at 50 °C (Figure S26).

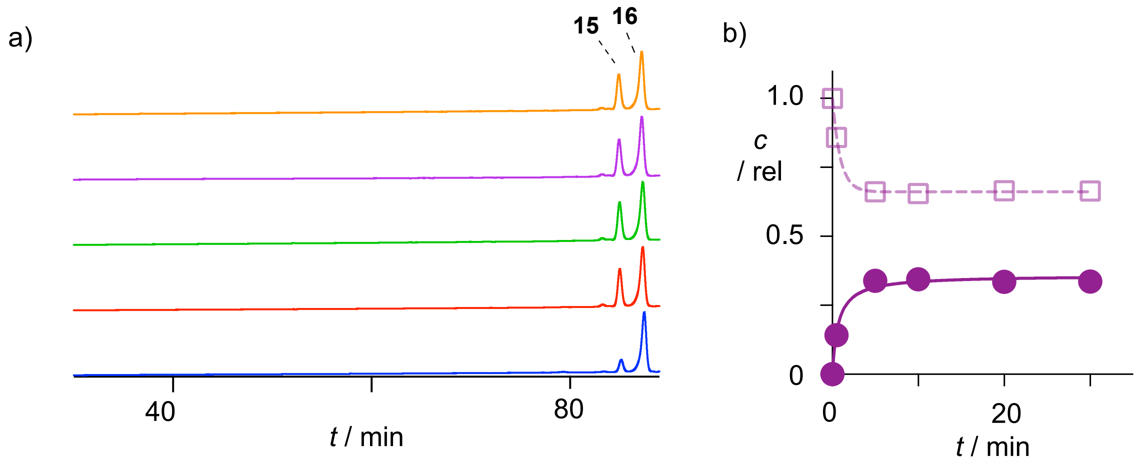

Figure S25. Reduction potential measurement of **16** at 50 °C. (a) HPLC traces (1 mL/min, from 0 to 15 min: H_2O, from 15 to 90 min: linear gradient H_2O/CH_3CN (+ TFA 0.1%) from 100:0 to 70:30; monitored at 550 nm) of the reaction mixture at t = 0.1, 5, 10, 20, 25 (from bottom to top) mins. (b) Relative concentration of **15** (filled circles) and **16** (empty squares) over time.

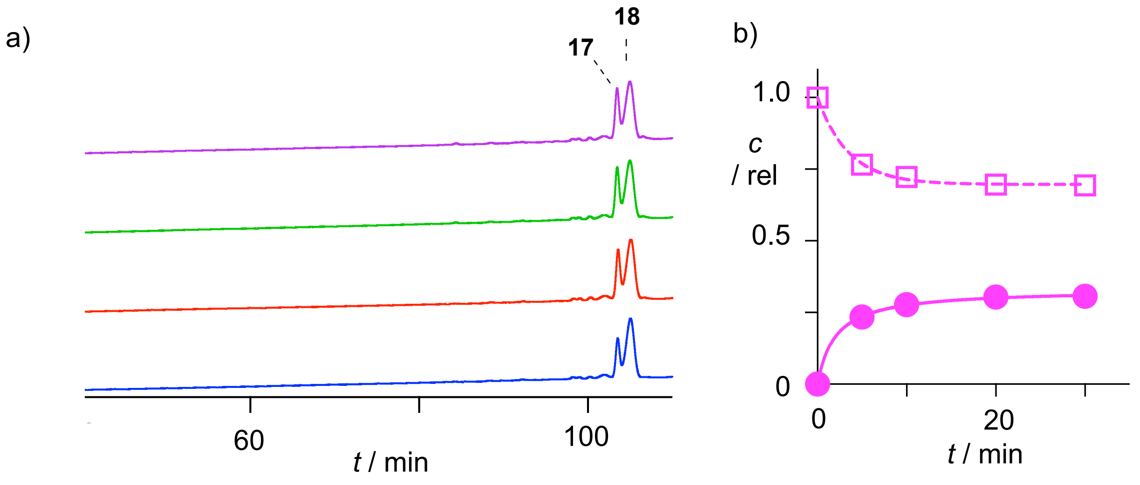

Figure S26. Reduction potential measurement of **18** at 50 °C**.** (a) HPLC traces (1 mL/min, from 0 to 15 min: H_2O, from 15 to 145 min: linear gradient H_2O/CH_3CN (+ TFA 0.1%) from 100:0 to 70:30; monitored at 550 nm) of the reaction mixture at t = 5, 10, 20, 25 (from bottom to top) mins. (b) Relative concentration of **17** (filled circles) and **18** (empty squares) over time.

Table S3. Summary of obtained K_{eq} and reduction potential values.[a]

Entry	SOI[b]	T [°C][c]	K_{eq}[d]	E_0 [mV][e]
1	Lipoic Acid[f]	25	16 ± 1	-290 ± 1
2	**9**	25	100 ± 10	-270 ± 2
3	**16**	25	3.3 ± 2.6	-320 ± 10
4	**16**	50	0.6 ± 0.3	-340 ± 10
5	**18**	25	0.05 ± 0.03	-370 ± 10
6	**18**	50	0.16 ± 0.05	-350 ± 5

[a]All measured in 10 mM phosphate buffer, pH = 7.3. [b]The substrate of interest was used in its oxidized form for reaction with reduced form of DTT. [c]Temperature of the reaction in Scheme S3, to determine the reduction potential. [d]Equilibrium constant calculated with Eq. S11. [e]Formal redox potentials, obtained with Eq. S12. [f]Data not shown, in agreement with previous literature (-288 mV).[S15] Data reported as the average of (at least) two independent replicates ± SEM.

10. Supporting References

[S1] G. Renno, D. Chen, Q.-X. Zhang, R. M. Gomila, A. Frontera, N. Sakai, T. R. Ward, S. Matile *Angew. Chem. Int. Ed.* **2024**, *63*, e202411347.

[S2] E. K. Bang, G. Gasparini, G. Molinard, A. Roux, N. Sakai, S. Matile, *J. Am. Chem. Soc.* **2013**, *135*, 2088–2091.

[S3] C. C. G. Scully, P. J. Rutledge, *Tetrahedron* **2010**, *66*, 5653–5659

[S4] Y. Cheng, L. Zong, J. López-Andarias, E. Bartolami, Y. Okamoto, T. R. Ward, N. Sakai, S. Matile, *Angew. Chem. Int. Ed.* **2019**, *58*, 9522–9526.

[S5] G. Gasparini, G. Sargsyan, E.-K. Bang, N. Sakai, S. Matile, *Angew. Chem. Int. Ed.* **2015**, *54*, 7328–7331.

[S6] Y. Cheng, A.-T. Pham, T. Kato, B. Lim, D. Moreau, J. López-Andarias, L. Zong, N. Sakai, S. Matile, *Chem. Sci.* **2021**, *12*, 626–631.

[S7] I. Shybeka, J. Maynard, S. Saidjalolov, D. Moreau, N. Sakai and S. Matile, *Angew. Chem. Int. Ed.* **2022**, *61*, e202213433.

[S8] W. B. Jin, C. Xu, Q. Cheng, X. L. Qi, W. Gao, Z. Zheng, E. W. C. Chan, Y.-C. Leung, T. H. Chan, K.-Y. Wong, S. Chen, K.-F. Chan, *Eur. J. Med. Chem.* **2018**, *155*, 285–302.

[S9] B. Lim, T. Kato, C. Besnard, A. I. Poblador Bahamonde, N. Sakai, S. Matile, *JACS Au* **2022**, *2*, 1105–1114.

[S10] S. Saidjalolov, X. -X. Chen, J. Moreno, M. Cognet, L. Wong-Dilworth, F. Bottanelli, N. Sakai, S. Matile, *JACS Au* **2024,** *4*, 3759–3765.

[S11] R. M. Martin, G. Tünnemann, H. Leonhardt, M. C. Cardoso, *Histochem. Cell Biol.* **2007**, *127*, 243–251.

[S12] J. C. Lukesh, M. J. Palte, T. Raines, *J. Am. Chem. Soc.* **2012**, *134*, 4057–4059.

[S13] J. G. Felber, L. Zeisel, L. Poczka, K. Scholzen, S. Busker, M. S. Maier. U. Theisen, C. Brandstädter, K. Becker, E. S. J. Arnér, J. Thorn-Sheshold, O. Thorn-Sheshold, *J. Am. Chem. Soc.* **2021**, *143*, 8791–8803

[S14] W. W. Cleland, *Biochemistry* **1964**, *3*, 480–482.

[S15] W. J. Lees, G. M. Whitesides, *J. Org. Chem.* **1993**, *58*, 642–647.

11. NMR Spectra

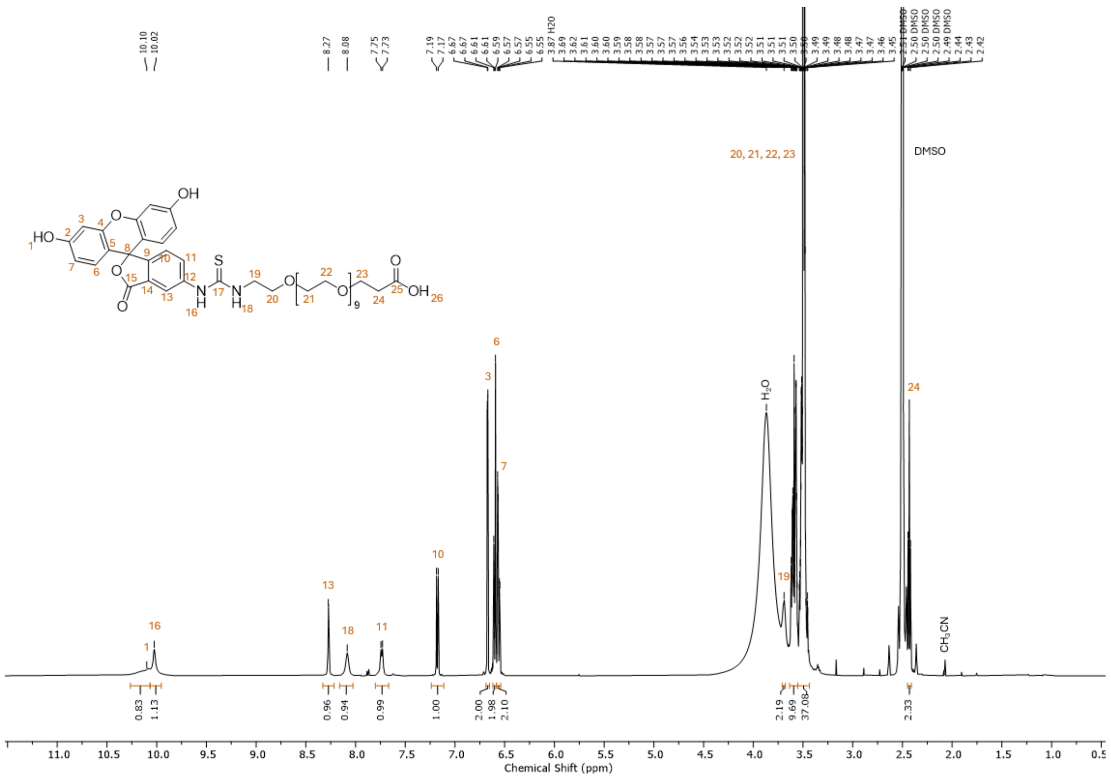

Figure S27. 500 MHz ^{1}H NMR spectrum (DMSO-d_6) of compound **3**.

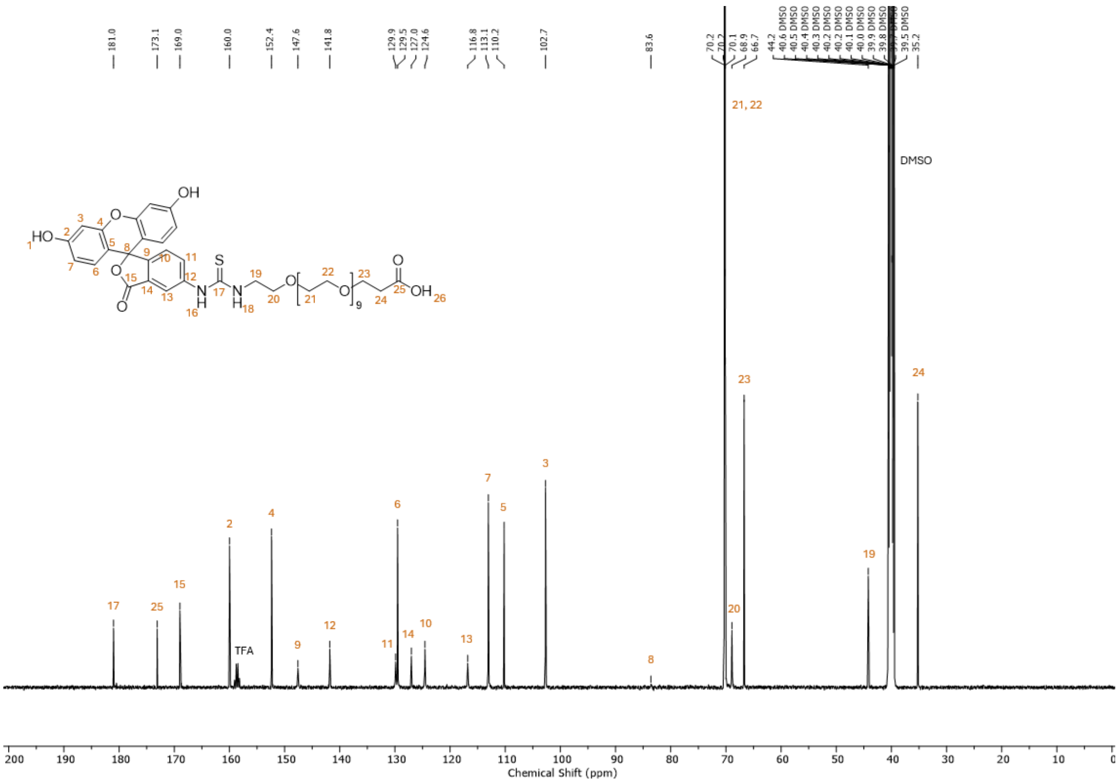

Figure S28. 126 MHz ^{13}C NMR spectrum of (DMSO-d_6) of compound **3**.

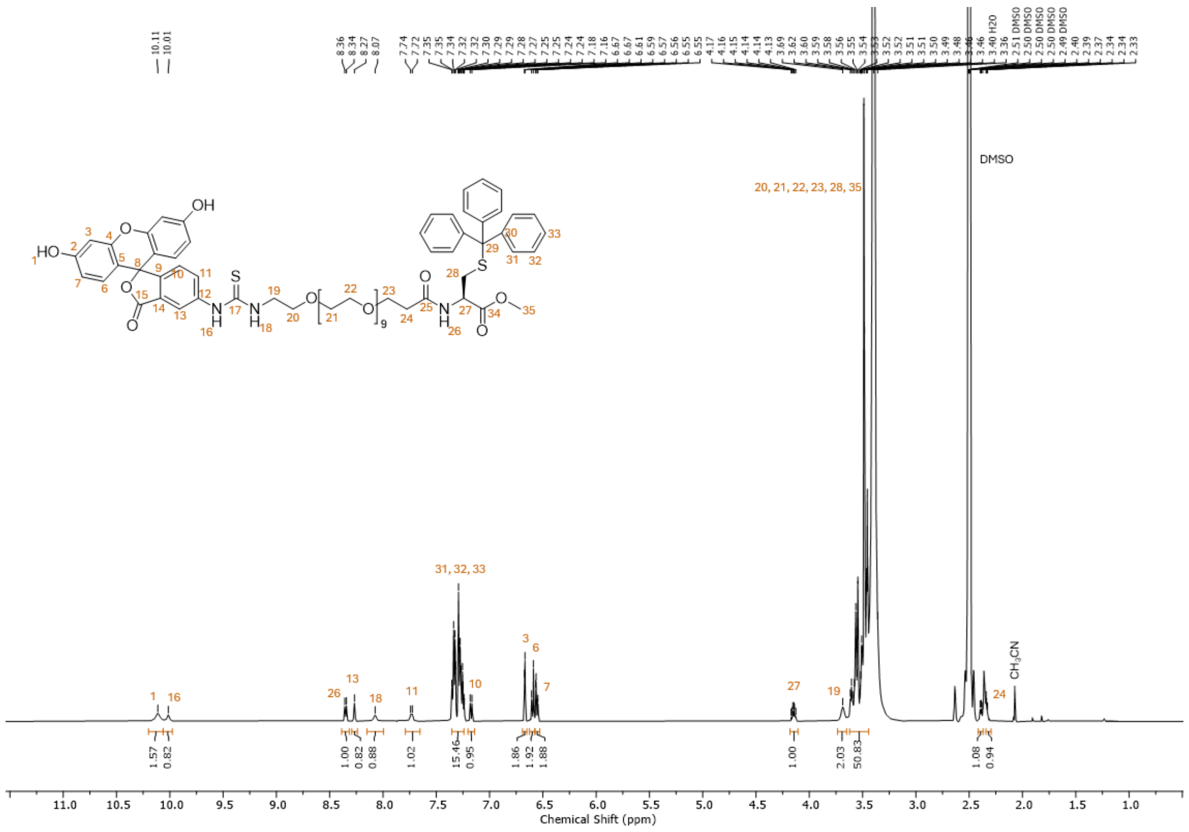

Figure S29. 500 MHz ^{1}H NMR spectrum (DMSO-d_6) of compound **27**.

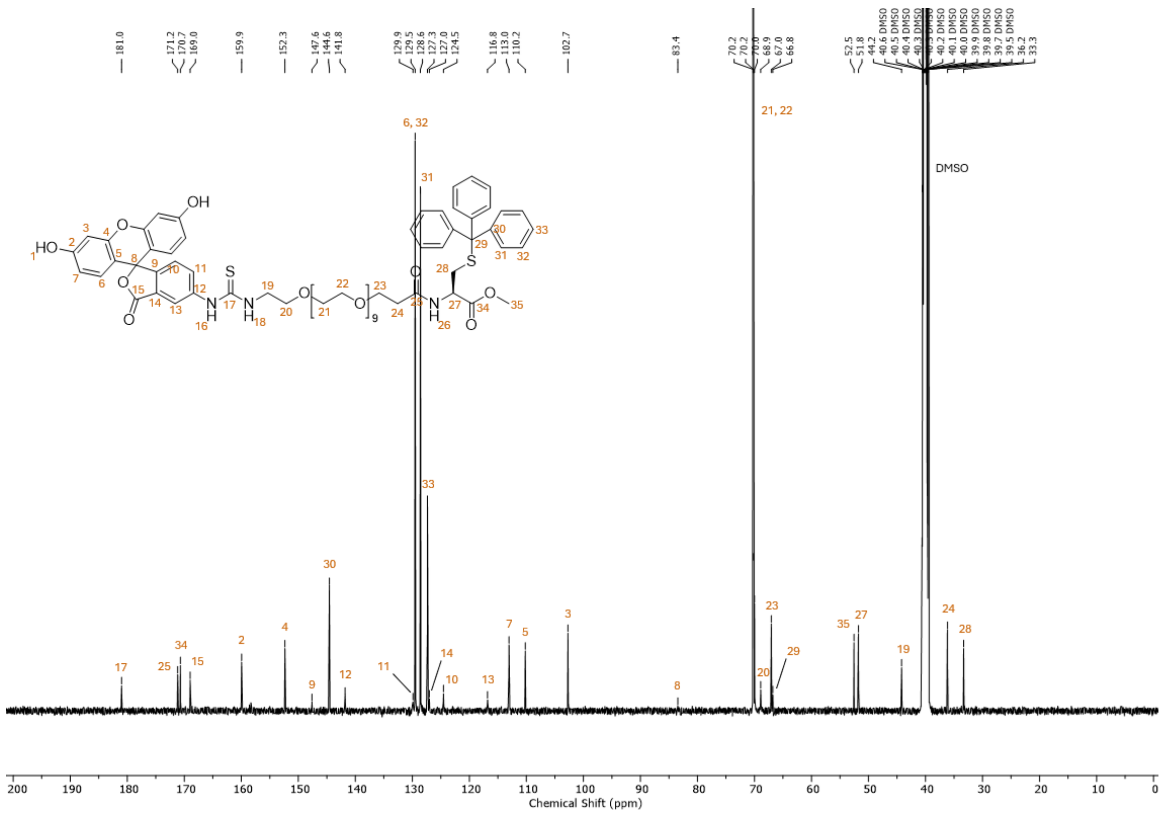

Figure S30. 126 MHz ^{13}C NMR spectrum (DMSO-d_6) of compound **27**.

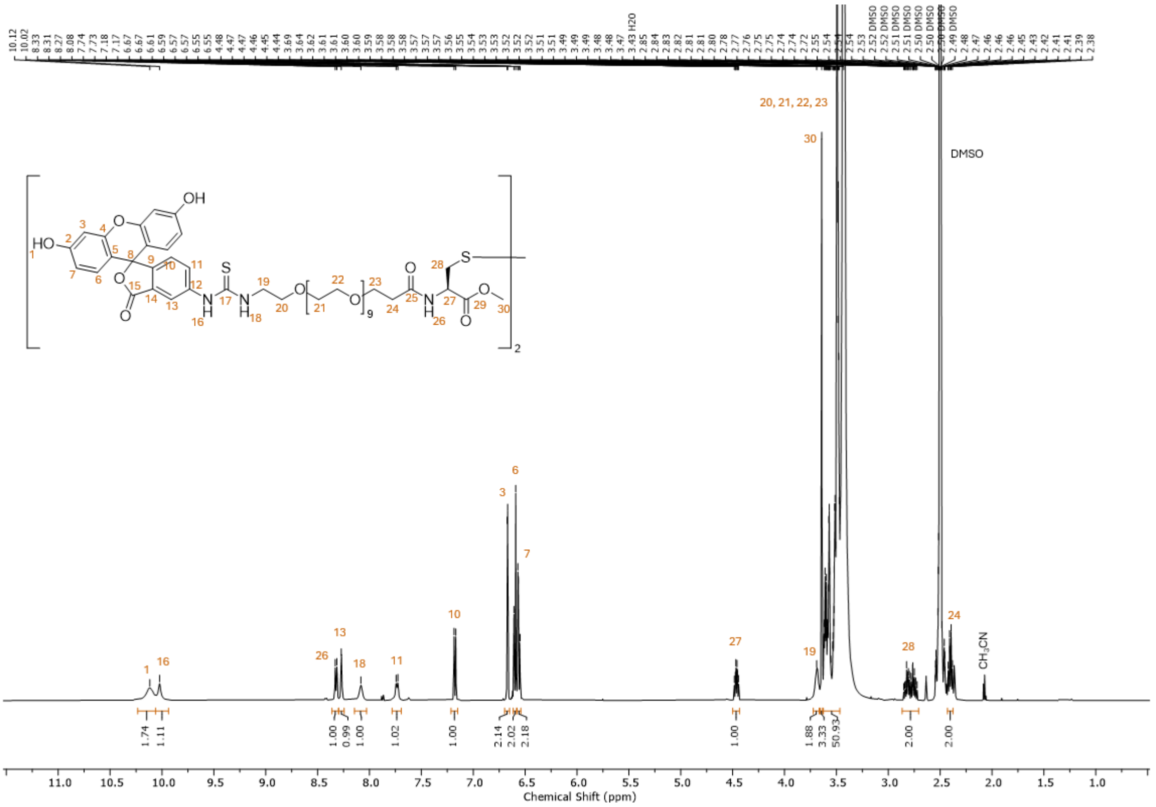

Figure S31. 500 MHz ^1H NMR spectrum (DMSO-d_6) of compound **28**.

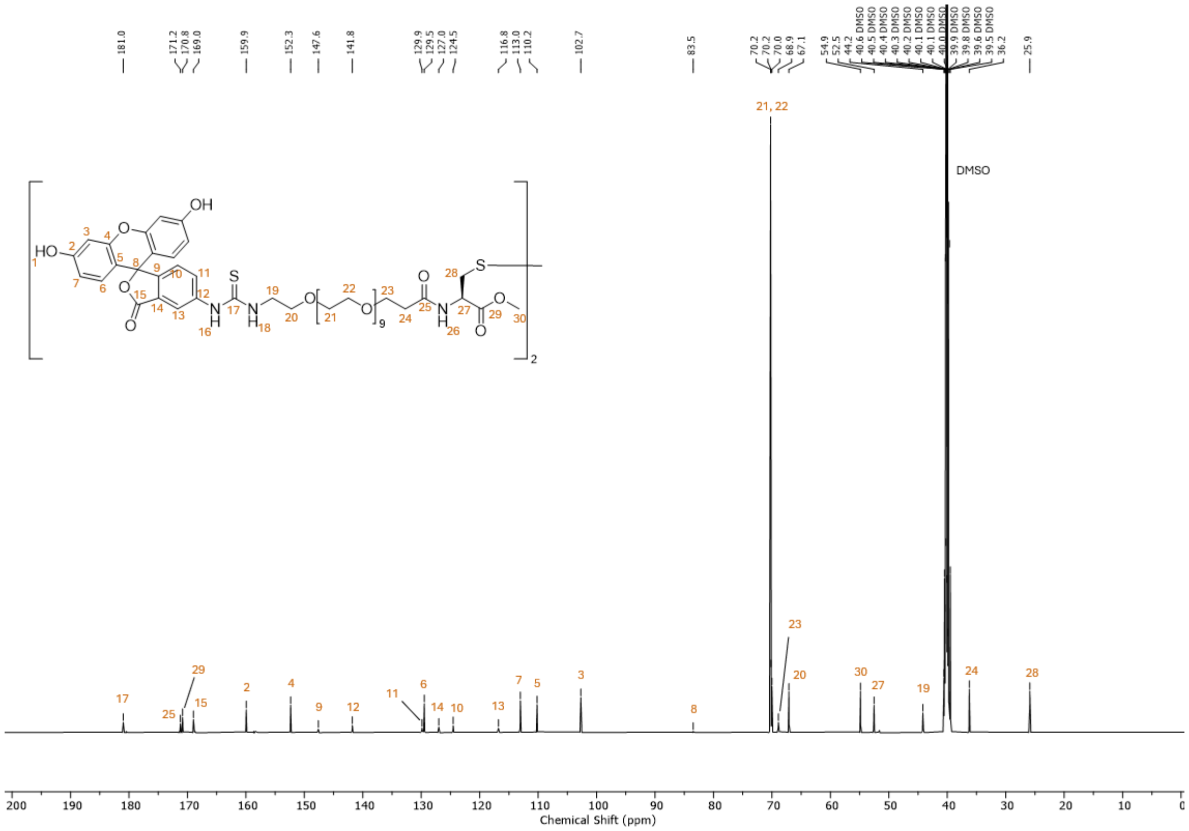

Figure S32. 126 MHz ^{13}C NMR spectrum (DMSO-d_6) of compound **28**.
